# Effect of targeted intervention on C-terminal agrin fragment and its association with the components of sarcopenia: a scoping review

**DOI:** 10.1007/s40520-023-02396-w

**Published:** 2023-03-28

**Authors:** Prabal Kumar, Kusumakshi Nayak, Shashikiran Umakanth, N. Girish

**Affiliations:** 1 Department of Physiotherapy, Manipal College of Health Professions, Manipal Academy of Higher Education, Manipal, Karnataka India; 2 Department of Medical Laboratory Technology, Manipal College of Health Professions, Manipal Academy of Higher Education, Manipal, Karnataka India; 3 Department of Medicine, Dr. TMA Pai Hospital, Udupi, Karnataka India

**Keywords:** Sarcopenia, Agrin, Biomarker, C-terminal Agrin Fragment

## Abstract

**Background:**

C-terminal Agrin Fragment (CAF) has emerged as a potent biomarker for identifying sarcopenia. However, the effect of interventions on CAF concentration and the association of CAF with sarcopenia components are unclear.

**Objective:**

To review the association between CAF concentration and muscle mass, muscle strength, and physical performance among individuals with primary and secondary sarcopenia and to synthesize the effect of interventions on the change in the level of CAF concentration.

**Methods:**

A systematic literature search was conducted in six electronic databases, and studies were included if they met the selection criteria decided a priori. The data extraction sheet was prepared, validated, and extracted relevant data.

**Results:**

A total of 5,158 records were found, of which 16 were included. Among studies conducted on individuals with primary sarcopenia, muscle mass was significantly associated with CAF levels, followed by hand grip strength (HGS) and physical performance, with more consistent findings in males. While in secondary sarcopenics, the strongest association was found for HGS and CAF levels, followed by physical performance and muscle mass. CAF concentration was reduced in trials that used functional, dual task, and power training, whereas resistance training and physical activity raised CAF levels. Hormonal therapy did not affect serum CAF concentration.

**Conclusion(s):**

The association between CAF and sarcopenic assessment parameters varies in primary and secondary sarcopenics. The findings would help practitioners and researchers choose the best training mode/parameters/exercises to reduce CAF levels and, eventually, manage sarcopenia.

**Supplementary Information:**

The online version contains supplementary material available at 10.1007/s40520-023-02396-w.

## Introduction

Sarcopenia, a term Irwin Rosenburg used in the late 1980s, means “loss of flesh” [1]. In the last two decades, a lot of focus has been given to understanding this condition, with various groups like the European Working Group on Sarcopenia in Older People (EWGSOP), Asian Working Group for Sarcopenia (AWGS), and International Working Group for Sarcopenia (IWGS) have come up with the definition which states sarcopenia as a condition characterized by gradual loss of skeletal muscle mass, loss of muscle strength, and reduced physical performance [2–4]. The prevalence of sarcopenia ranges from 9 to 12% in community-dwelling older adults, 23–24% among hospitalized people, and 30–50% among residents of long-term care settings, and its incidence increases with age [5, 6]. Considering the complex and multifactorial etiology and association with multiple adverse events, the early identification of sarcopenia is of utmost importance to promote healthy aging [7–12].

Despite the criteria set by EWGSOP and AWGS to assist in assessing sarcopenia [2, 3], challenges with acquiring valid performance and the amount of muscle data continue to obstruct the consistent implementation of diagnostic and therapeutic programs [13, 14]. While techniques like dual-energy X-ray absorptiometry (DEXA), magnetic resonance imaging (MRI), computed tomography (CT), and bioimpedance analyzer (BIA) can quantify lean mass but are expensive and difficult to use in different clinical settings like community settings, and primary health centers [13, 15, 16]. Furthermore, researchers debate the objectivity of muscle mass, muscle strength, and physical performance evaluations in older adults, as these are performance-oriented and demand active involvement [14, 17]. As a result, direct methods of assessing muscle mass and performance for the older population are only sometimes practical. Lack of motivation, pain, and depressed mood could interfere with the testing, which might not produce plausible results [17]. Hence, as a sarcopenia screening method, a blood-based biomarker could be a more accessible choice [14].

The search for sarcopenia biomarkers that provide additional information obtained from clinical data has become especially important in recent years. Ascertaining the pathophysiology of the disease is key to the development of sarcopenia biomarkers. The pathogenesis underlying the onset and development of sarcopenia is multifactorial [18–20]. The importance of the neurophysiological process in maintaining skeletal muscle health with advancing age has recently gained credence [21, 22]. Reduced reinnervation capacity due to age-related disruption at the neuromuscular junction (NMJ) is recognized as a significant contributor to the development and progression of sarcopenia [23]. In fact, as a person ages, considerable remodeling occurs in NMJ, which is essential for the nerve to muscle cross-talk [21]. As neuromuscular remodeling occurs, the neuronal protease neuro-trypsin proteolytically cleaves and inactivates Agrin, a well-established mediator of NMJ formation and stabilization, dissociating a 22-kDa C-terminal Agrin Fragment (CAF) [24], easily quantifiable in human blood. As a result, the post-synaptic acetylcholine receptor clustering will be compromised, which will cause a gradual buildup of denervated muscle fibers [21]. In light of this, biomarkers of NMJ stability have been suggested as early signs of sarcopenia [25].

The circulatory level of CAF may represent an early indicator of NMJ dismantling and muscle fiber denervation, which signals the onset of sarcopenia [14, 21]. A study on healthy older adults reported that the serum CAF concentration was significantly related to the onset of neuromuscular fatigue [26]. On the other hand, older persons who engage in aerobic activities and balance training reported significantly higher mobility and correspondingly lower CAF levels [27–29]. Thus, a change in the concentration of CAF following therapeutic exercise would be a relevant outcome measure along with the assessment of muscle mass, muscle strength, and physical performance, which are performance-oriented.

The wide availability and the low cost of CAF reinforce the potential for use in daily clinical practice by allowing identification or at least contributing to the early identification of patients at risk of sarcopenia in a simple way while requesting a routine blood test [30, 31]. Understanding the CAF association with components of sarcopenia (muscle mass/muscle strength/physical performance) is also of great interest for developing, adapting, or evaluating therapeutic exercise programs and prophylactic treatments. However, a scoping review summarizing and discussing the association between CAF and components of primary and secondary sarcopenia, as well as comparing the effect of different interventions on the level of CAF concentration among sarcopenic older adults, is lacking. The review would be a valuable resource for physicians and researchers who intend to include CAF in research studies and clinical practice as a biomarker of sarcopenia. Thus, this review aims to scope the scientific evidence about the association between the level of CAF and primary sarcopenia, the level of CAF and secondary sarcopenia, and the effect of various interventions (exercise/nutrition/hormonal) on the change in the level of CAF among older adults. We hypothesized that the higher CAF levels would be associated with primary and secondary sarcopenia, and the interventions (exercise/nutrition/hormonal) would reduce the level of CAF among older adults.

## Methods

Scoping review methodology was chosen as it is the most appropriate method to identify the key characteristics related to the topic of investigation: the association between CAF and primary sarcopenia, the association between CAF and secondary sarcopenia, and the effect of interventions (exercise/nutrition/hormonal) on the levels of the CAF [32]. This review followed the Joanna Briggs Institute (JBI) Scoping Review Methodology [33] and Preferred Reporting Items for Systematic reviews and Meta-Analysis extension for Scoping Reviews (PRISMA-ScR) checklist was used for reporting processes [34].

### Data sources and search strategy

A systematic literature search was undertaken from April to September 2022 on the following six electronic databases: PubMed, Scopus, Embase, Cumulated Index to Nursing and Allied Health Literature (CINAHL), ProQuest, and Web of Science. Relevant MeSH terms and Boolean phases used for the search: “CAF” OR “agrin” OR “neuromuscular junction degeneration” OR “agrin receptor” OR “blood biomarker” AND “Sarcopenia” OR “physical functional performance” OR “hand strength” OR “muscle, skeletal” AND “older adult” OR “aged” OR “senior citizens” without time restriction and age/ humans as filters were applied (Supplementary material 1).

### Eligibility criteria

The studies were included if they met the following criteria: (a) original study, (b) on older adults, (c) including those diagnosed with primary or secondary sarcopenia with specific diseases, e.g., chronic kidney disease, stroke, Parkinson’s, Chronic Obstructive Pulmonary Disease (COPD), and congestive heart failure, (d) any study design, (e) studies looking at the effect of a different intervention (exercise/hormonal/nutrition) on CAF level, and (f) studies analyzing the association between CAF and sarcopenia and/or its outcome measures like muscle mass, muscle strength, and physical performance. The studies were excluded if they failed to meet the inclusion criteria and/or: (a) full text was not available, (b) language was other than English, (c) provided no extractable data, (d) studies not included CAF biomarker, and (e) animal studies.

### Selection process

Two reviewers, PK and GN, independently searched the literature. The identified studies were imported to Rayyan (Ref. # 488,637) software. After resolving the duplicates, two reviewers (PK and GN) conducted title and abstract screening separately. If the study was deemed suitable, it progressed to retrieving the full text. Any conflict regarding the study selection was resolved by discussion with two reviewers, KN and SU. PK did a full-text reading, and data extraction was carried out from the relevant studies.

### Data charting process and data items

Four included studies were randomly selected and shared with all the reviewers, and the data charting sheet was prepared individually and piloted. Further, the data charting sheet was finalized after a consensus discussion with all the authors. The two authors extracted the data in duplicate for each retrieved article. Any disagreement between the two reviewers on the extracted data from the documents was resolved by simultaneously analyzing the data. For relationship studies between CAF and primary sarcopenia, Inclusion criteria, Exclusion criteria, Sarcopenia diagnostic criteria, Muscle mass, Muscle strength, Physical performance, Statistical analysis, and Association between primary sarcopenia and CAF were extracted; for relationship studies between CAF and secondary sarcopenia, Inclusion, Exclusion, Co-morbidity(ies), Sarcopenia diagnosis criteria, Muscle mass, Muscle strength, Physical performance, Statistical analysis, and Association between secondary sarcopenia and CAF were extracted; for studies which have evaluated the effect of interventions (exercise/nutrition/hormone), Randomization, Intervention, Exercise parameters, Control group, Supervision & Motivation strategies, Outcome measures, Blood collection, CAF Values, Results of relationship and Findings were extracted.

### Critical appraisal of individual sources of evidence

Critical appraisal of the individual studies finding out the relationship of CAF and sarcopenia and/or sarcopenia with associated co-morbidities was done by two authors, PK and GN, using JBI critical appraisal checklist for cross-sectional studies [35]. The Consensus on Exercise Reporting Template (CERT) [36] was used to evaluate the completeness of exercise reporting the studies included in this review. The Physiotherapy evidence database scale (PEDro Scale) was used to assess the methodological quality (risk of bias) of the intervention studies included in the review [37].

## Results

### Search results

**Fig. 1:**
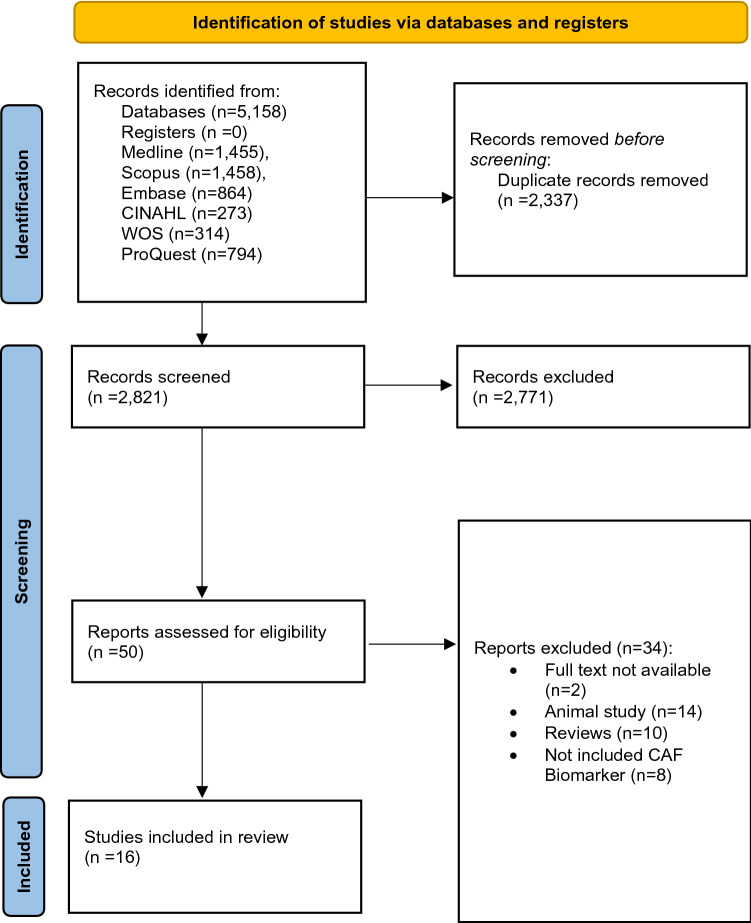
Preferred reporting items for systematic review and meta-analysis statement 2020 (PRISMA 2020) flow diagram

A total of 5,158 studies were identified through database searches. After removing duplicate studies (*n* = 2337), the titles and abstracts of 2821 studies were screened. A review of the titles and abstracts yielded 50 relevant studies for full-text screening. Finally, 16 studies met all inclusion criteria and were included in this review. A Preferred Reporting Items for Systematic Review and Meta-analysis 2020 (PRISMA 2020) flowchart of the literature search depicts the screening process (Fig. 1) [38].

The results have been taxonomized in three sections: the association between CAF and primary sarcopenia, the association between CAF and secondary sarcopenia, and the effect of interventions (exercise/nutrition/hormonal) on the levels of the CAF.

### Association between levels of CAF and primary sarcopenia

A total of four (*n* = 4) studies have been found assessing the association between the levels of CAF and primary sarcopenia [14, 25, 29, 39]. These studies adopted different designs—one each (*n* = 1) was cross-sectional [29], multi-center non-randomized trial [25], randomized trial [39], and cohort designs [14]. The criteria used to find out the sarcopenia among the participants varied in the studies, with one (*n* = 1) study using EWGSOP criteria [25]; two (*n* = 2) studies utilized the criteria forwarded by EWGSOP2 [14, 29]; while two (*n* = 2) studies have not reported the criteria used [39]. Looking at the triad of muscle mass, muscle strength, and physical performance to confirm sarcopenia, two (*n* = 2) studies have measured the physical performance [29, 39], three studies (*n* = 3) have assessed the grip strength [14, 25, 29], and three (*n* = 3) studies have assessed the muscle mass [14, 29, 39].

The procedure followed for blood sample collection has been described in the studies. Among the four (*n* = 4) studies, two (*n* = 2) have clearly stated and mentioned the median cubital vein as a source to collect the sample [14, 29]. The 5 ml amount of blood drawn has been reported by one (*n* = 1) study [29]. Two (*n* = 2) studies mentioned − 80 degree Celsius as the preferred temperature to store the collected blood until future analysis [14, 39]. Three studies (*n* = 3) have used serum to measure the levels of CAF concentrations [25, 29, 39], and only one (*n* = 1) used plasma [14]. The studies have used different procedures to analyze the CAF concentration levels. Two (*n* = 2) studies have utilized the western blot [25, 39], and two (*n* = 2) have used the commercially available (Enzyme-Linked Immunosorbent Assay) ELISA kit (Abcam #ab216945) [14, 29].

Three studies (*n* = 3) have reported a significant association between CAF and sarcopenia (*p* value ranging from < 0.01 to < 0.00001) [14, 25, 29]. Two studies (*n* = 2) have reported the association between appendicular lean mass (ALM) and the level of CAF [14, 39]. These studies have reported a strong inverse relationship (male: *r* =  − 0.524; female: *r* =  − 0.219) and significant association (*p* = 0.003) between ALM and = CAF levels, respectively. The association between the hang grip strength (HGS) and CAF has been reported by two (*n* = 2) studies [14, 39], one stating low HGS displayed significantly higher levels of CAF (*p* = 0.027) while the other found no association (Table 1). Among the two (*n* = 2) studies that assessed physical performance, one study (*n* = 1) reported gait speed not associated with CAF concentration [39]. However, the study by Marcolin et al. 2021 showed that active older dancers (AOD) having low CAF concentration have better performance in TUG (*p* < 0.001), 10-MWT (*p* < 0.001) compared to the sedentary older [29].

**Table 1:** Association between levels of CAF and primary sarcopenia

Author, year, study design	Title	Objective	Sample population	Inclusion criteria	Exclusion criteria	Sarcopenia diagnosis criteria	Blood sample CAF (Serum/plasma) CAF analysis	Muscle mass	Muscle strength	Physical performance	Statistical analysis	Association between CAF and primary sarcopenia
Hettwer et al. 2013 Multi-center, non-randomized, open-label, clinical study	Elevated levels of a C-terminal agrin fragment identifies a new subset of sarcopenia patients	To ascertain the significance of elevated agrin degradation in the development of human sarcopenia	Initial screening *n* = 750 *n* = 350 were eligible for a secondary screening Sarcopenic (SP group) *n* = 73 subjects (34 women and 39 men, ages 65 to 87) Aged matched control (AMC group) *n* = 60 subjects (28 women and 32 men, ages 65 to 88 years)	SP group: DEXA scan values below − 1 (SD) AMC group: DEXA values > 0	Comorbidities, such as diabetes, COPD, renal difficulties, osteoporosis, and other medical ailments	EWGSOP	Taken the blood of consenting healthy Swiss blood donors (BD group) Serum Western blot	Total body mass: by scale BMI	Grip strength: Isometric Jamar dynamometer from JLW Instruments, Chicago Knee strength: maximal isokinetic strength of knee extensors by a Kin-Com 125 AP dynamometer (Chattanooga, TN)	NA	Data were statistically analyzed with the Student's t test and using the free statistic tool R (version 2.9.0) Scatter plots between CAF concentration and age are shown for the BD group for both sexes and for the AMC and SP groups	Average CAF level of the SP group is highly significantly elevated compared to the age-matched control group AMC (*p* < 0.00001) SP group: Tendency for age-associated elevated CAF levels SP group: Values were significantly higher with 4.18 ± 2.30 ng/ml for females and 5.17 ± 2.79 ng/ml for males (the standard deviation was larger than in both other control groups with no significant gender difference)
Drey et al. 2013 Participants were drawn from a randomized, controlled, single-blinded trial	C-terminal Agrin Fragment as a potential marker for sarcopenia caused by degeneration of the neuromuscular junction	To evaluate relevant influencing variables on the concentration of cleaved C-terminal Agrin Fragments in the serum of prefrail community-dwelling older adults	69 prefrail participants, aged between 65 and 94 years, were randomized into three arms: 1. “power training” (n = 24), 2. “strength training” (n = 23) and 3. “control group” (n = 22)	Pre-frail older adults as per Fried and colleagues criteria	Depression (GDS 1 5), dementia (MMSE: 25), BMI: 35 kg/m 2, intake of immunosuppressive drugs, history of kidney stones, sarcoidosis, plasmacytoma, COPD, inflammatory bowel disease, angina pectoris, history of cancer and current attendance of muscle training	Fried and colleagues	Serum sample were stored in 2 ml screw-cap polypropylene tubes and frozen at -80 degrees Celsius Serum Western blotting	Body composition: appendicular lean mass (aLM): DEXA	Hand grip strength	Gait speed	Scatter plots between CAF concentration and aLM and CAF concentration and age for both sexes Degree of correlation: Pearson’s correlation coefficient Multiple linear regression models: Association between CAF concentration and aLM standardized regression coefficients of the multiple linear regression models at baseline (time point 0, T0)	aLM significantly explanatory factor for CAF concentration Correlation between CAF concentration and aLM: male show strong correlation: r = − 0.524, p = 0.015; while female not: r = − 0.219, p = 0.140) Hand grip strength not associated with CAF concentration Gait speed not associated with CAF concentration
Pratt J et al. 2021 Cross-sectional cohort study	Plasma C-Terminal Agrin Fragment as an Early Biomarker for Sarcopenia: Results From the GenoFit Study	To examine the indicative relevance of plasma CAF concentrations to sarcopenic phenotypes in a large sample of healthy older adults	*n* = 300 aged between 50 and 83 years were randomly selected from a pool of 1728 individuals aged ≥ 50 years (males, *n* = 150; mean age: 64.2 ± 8.7 years and females, *n* = 150; mean age: 63.9 ± 8.3 years)	Aged ≥ 50 years, be without renal function abnormalities, be free from any chronic disease or musculoskeletal injury and be able to provide informed consent	Not following inclusion criteria were excluded	EWGSOP2 criteria	Blood samples were obtained by venepuncture of the median cubital vein using vacutainers containing an DTA anticoagulant (BD Vacutainer) For plasma separation, samples were centrifuged at 4000 g for 10 min at 4 °C and the extracted plasma was stored in aliquots at − 80 °C until analysis Plasma CAF Commercially available ELISA kit (Abcam #ab216945)	ALM: DEXA (Lunar Prodigy, GE Healthcare Technologies, Chicago, IL)	Grip strength: Jamar digital hand-held dynamometer (JLW Instruments, Chicago, IL)	NA	Independent-sample Student’s t test was used to assess differences between population characteristics according to the presence of sarcopenia Multiple linear regression models: Association between CAF concentrations, ALM, and grip strength Analysis of covariance: Compare mean plasma CAF levels between sarcopenic domains Receiver operating characteristic (ROC) curve analysis: The utility of plasma CAF for diagnosing sarcopenia, low ALM, and low grip strength	Compared to non-sarcopenic controls (2.7 ± 0.5 ng/ml), sarcopenic individuals (3.1 ± 1.4 ng/ml) had significantly elevated plasma CAF concentrations (p < .001) Multiple regression model adjusted for confounders, such as sex, age, and BMI: CAF concentration were significantly associated with ALM (*p* = 0.28), but not grip strength (*p* = 0.575) Plasma CAF concentration was significantly higher in individuals with low grip strength (*p* = 0.027) or low ALM (*p* = 0.003), when compared to those with normal grip strength or ALM Overall, individuals with low grip strength or low ALM displayed significantly higher CAF levels compared to healthy controls, after adjusting for age and BMI (*p* = 0.027, *p* = 0.003, respectively) In males, those with low grip strength or low ALM had significantly elevated CAF levels (*p* = 0.039, *p* = 0.027, respectively) In females, only those with low ALM had significantly raised CAF concentration, compared to healthy controls (p = 0.035)
Marcolin et al. 2021	Active older dancers have lower C-terminal Agrin fragment concentration, better balance and gait performance than sedentary peers	To investigate the beneficial effects of regular practice of dancing in physically active elders on concentration of C-terminal Agrin fragment (CAF), a marker of NMJ instability, muscle mass, strength, and physical performance	Thirty-one healthy older adults (19 females and 12 males; age: 70.5 ± 4.9 yr; height: 1.64 ± 0.08 m; body mass: 72.1 ± 11.2 kg) were enrolled for the study *n* = 16 active older dancer (AOD): 2.2 ± 1.01 h/week dance practice besides light aerobic physical activities (such as walking, or cycling) *n* = 15 older sedentary (OS): not involved in any type of physical activity at time of study	No history of orthopedic injuries and no falls in the last year, absence of neurological pathologies and non-corrected sight or vestibular disorders	NR	EWGSOP 2 criteria	Blood sampling of 5 ml from the median cubital vein Blood samples were centrifuged at 3000 Rpm for 10 min to separate serum from the other blood's component Serum Commercially available enzyme-linked immunosorbent assay (ELISA) kits (Human Agrin Simple Step ELISA, Ab216945, Abcam, Cambridge, United Kingdom)	Muscle morphology of vastus lateralis: Ultrasound scan Cross-sectional area (CSA) of Rectus femoris, VL, Multifidus	Handgrip test: Digital hand dynamometer (Jamar, JLW Instruments, Chicago, IL, USA) Isometric strength of the knee extensor	TUG 10-m walk test (10-MWT)	Two-tailed unpaired t-tests were carried out to compare walking speed, upper and lower limb strength, muscle morphology and concentration of circulating CAF between the two groups of participants One way ANOVA: Compare the concentration of circulating CAF of both OS and AOD with those of young reference control group For each dependent variable Cohen's d was also calculated Pearson’s correlation: Relationship between the concentration of circulating CAF and functional parameters	Only two OS participants and three AOD participants were classified as pre-sarcopenic The blood analysis showed higher CAF concentration in the OS (5259 ± 941 pg/ml) compared to AOD (4389 ± 718 pg/ml) and this difference was statistically significant (*p* < 0.01; t = 2.812; df = 27; Cohen’s *d* = 1.04) Compared to the young reference control group, the one-way ANOVA showed statistically significant differences among the three groups (*F* = 20.63; ηp^2^ = 0.496; *p* < 0.0001) Specifically, the OS had CAF concentration 1.5-fold higher (*p* < 0.01) whereas the AOD had CAF values 1.28-fold higher (p = 0.012)

*SP* sarcopenic, *AMC* aged-matched control, *COPD* chronic obstructive pulmonary disease, *EWGSOP* European working group on sarcopenia in older people, *CAF* C-terminal agrin fragment, *BMI* body mass index, *GDS* geriatric depression scale, *MMSE* mini-mental state examination, *DEXA* dual-energy X-ray absorptiometry, *ALM/aLM* appendicular lean mass, *EDTA* ethylenediaminetetraacetic acid, *ROC* receiver operating characteristic curve, *10-MWT* 10-m walk test, *AOD* active older dancers, *OS* older sedentary

### Association between levels of CAF and secondary sarcopenia

A total of six (*n* = 6) studies have reported the relationship between levels of CAF and sarcopenia in individuals with co-morbidities [17, 30, 40–43]. Among the included six studies, two studies (*n* = 2) are cohort studies [42, 43], and four (*n* = 4) studies are cross-sectional in design [17, 30, 40, 41]. The co-morbidities among the individuals were stroke (*n* = 1) [42], COPD (*n* = 1) [41], heart failure (*n* = 1) [43], Parkinson’s (*n* = 1) [40], and hip fracture (*n* = 1) [17], with one study (*n* = 1) included individuals with multi-morbidity [30].

The sarcopenia criteria used among the studies vary, with two studies (*n* = 2) have used EWGSOP as criteria [17, 30], two (*n* = 2) have used EWGSOP2 [40, 41], one (*n* = 1) used appendicular skeletal muscle mass [43], and one (*n* = 1) study has not reported the criteria used to assess the sarcopenia [42]. The muscle mass has been evaluated in all the studies, with four studies (*n* = 4) have used a BIA [17, 40–42], one study (*n* = 1) used DEXA [43], and one study (*n* = 1) used mid-arm circumference using inch tape to assess the muscle mass [30]. The HGS has been evaluated in five studies (*n* = 5), with three studies (*n* = 3) used a hand-held hydraulic dynamometer [17, 30, 42], and two studies (*n* = 2) used a digital hand-held dynamometer [40, 41]. Only one study (*n* = 1) has not assessed muscle strength [43]. The third component, physical performance, of the sarcopenia assessment triad, has been assessed in four studies (*n* = 4), with two studies (*n* = 2) used short physical performance battery (SPPB) [40, 41] and one each (*n* = 1) assessing physical performance using a six-minute walk test (6-MWT) [43] and 4-m walk test [30], respectively.

The details of the method used to draw the blood sample have been reported by four studies (*n* = 4) [17, 30, 42, 43]. In two studies (*n* = 2), method used to draw the blood sample has not been reported in detail [40, 41]. Three studies (*n* = 3) mentioned clearly that the participants were asked to remain overnight fast before their blood samples were taken [17, 30, 42]. Among the included studies, four (*n* = 4) used the serum [17, 30, 42, 43], while two (*n* = 2) used the plasma [40, 41]. All six studies (*n* = 6) have mentioned that the CAF levels were analyzed using commercially available ELISA kits (NTCAF, ELISA, Neurotune, Schlieren-Zurich, Switzerland).

The level of CAF concentration was significantly higher among secondary sarcopenics (*p* value range from < 0.05 to 0.0001). Two studies (*n* = 2) reported a significant association between CAF levels and muscle wasting [30, 43]. Four studies (*n* = 4) reported a significant association between the level of CAF and reduced physical performance, with one study (*n* = 1) reporting that individuals with less than 400-m distance covered in 6-MWT have a higher concentration of CAF (*p* = 0.0001) [43], two studies (*n* = 2) reported the speed < 0.8 m/sec in 4-m walk test have a higher CAF concentration (*p* value range < 0.003 – < 0.001) [30, 43], and two studies (*n* = 2) reported higher concentration in an individual with less score in SPPB (9–10 score, *p* < 0.01; 6–8 score, *p* < 0.01) [40, 41]. A significant negative association ( *p* < 0.001) has been reported between CAF and HGS in two (*n* = 2) studies [30, 40]. Two (*n* = 2) studies have reported a reduction in CAF levels following rehabilitation which were associated with improvement in hand grip strength (*p* value range < 0.05 – 0.01) [41, 42] (Table 2).

**Table 2:** Association between levels of CAF and secondary sarcopenia

Author, Year, study design	Title	Objective	Sample population	Inclusion criteria	Exclusion criteria	Co-morbidity	Sarcopenia diagnosis criteria	Blood sample CAF (Serum/plasma) CAF analysis	Muscle mass	Muscle strength	Physical performance	Statistical analysis	Association between CAF and secondary sarcopenia
Marzetti et al. 2014	Serum levels of C-terminal agrin fragment (CAF) are associated with sarcopenia in older hip fractured patients	To verify if serum CAF levels were associated with sarcopenia in a cohort of older adults hospitalized for traumatic hip fracture	*n* = 42 hip fractured patient	Older adults admitted for hip fracture due to accidental fall to the emergency department	Age < 65 years Presence of peripheral edema Bone metastasis Cognitive impairment (Cognitive performance scale, CPS < 3) Presence of pacemaker or implantable cardioverter defibrillator unwillingness to take part	Hip fracture	EWGSOP	Blood samples were obtained in the morning by venepuncture of the median cubital vein after overnight fasting within 24 h from ED admission, using commercial collection tubes (BDMedical, Franklin Lakes, NJ) Samples were left at room temperature for 20 min and subsequently centrifuged at 1000 × g for 10 min at 4 °C. The supernatant, corresponding to the serum fraction, was aliquoted and stored at − 80 °C until analyses Serum Commercial ELISA kit (NTCAF ELISA, Neurotune AG, Schlieren—Zurich, Switzerland)	Skeletal Muscle Index (SMI): BIA	Muscle strength: North Coast hand-held dynamometer (North Coast, CA)	NA	Analysis of covariance (ANCOVA) was used to examine the relationship between serum CAF levels and the presence of sarcopenia Variables considered for adjustment were those showing a significant difference between sarcopenic and non-sarcopenic subjects at the univariate analysis	Serum levels of CAF were significantly higher in patients with sarcopenia compared with non-sarcopenic subjects (*p* < 0.001) Association remained significant in male (sarcopenic and non-sarcopenic, *p* = 0.04) and female (sarcopenic and non-sarcopenic, *p* = 0.02)
Steinbeck et al. 2015 Cohort study	Detection of muscle wasting in patients with chronic heart failure using C-terminal agrin fragment: Results from the Studies Investigating Co-morbidities Aggravating Heart Failure (SICA-HF)	To study the diagnostic properties of CAF to identify skeletal muscle wasting in patients with HF	All (*n* = 196) Muscle wasting (*n* = 38) No muscle wasting (*n* = 158)	Diagnosis of heart failure and either a left ventricular ejection fraction ≤ 40% or a left atrial dimension > 4.0 cm (> 2.5 cm/m height), or N terminal pro-brain natriuretic peptide (NT-proBNP) > 400 pg/ml or BNP > 150 pg/mL, aged > 18 years and be willing to provide informed consent	Patients with previous heart transplantation, a history of unstable angina, myocardial infarction, stroke, cardiovascular revascularization, or open abdominal surgery within 6 weeks of the planned baseline visit, known pregnancy, haemodialysis at baseline, or unable to understand and comply with protocol or to give informed consent	Heart failure	Appendicular skeletal muscle mass two standard deviations below the mean of a young healthy reference group aged 18–40 years	Patients’ blood was drawn from an antecubital vein in fasting condition and CAF were analyzed from serum samples immediately centrifuged and frozen at − 80 ∘C until analysis Serum A sandwich enzyme-linked immunosorbent assay (ELISA (NTCAF ELISA and NTtotalCAF ELISA; Neurotune, Schlieren-Zurich, Switzerland) was used to detect CAF	DEXA scan	NA	6-MWT	Serum levels of CAF were non-normally distributed and therefore log-transformed to achieve a normal distribution Analysis of variance (ANOVA), Student’s unpaired *t test*, Fisher’s exact test, Pearson’s simple regression, and logistic regression were used as appropriate MedCalc for Windows version 11.2.1.0 (Broekstraat, Mariakerke, Belgium) was used to perform receiver operating characteristics (ROC) curve analysis Areas under the ROC curve (AUCs) were constructed for sensitivity and specificity to compare different predictive values	Levels of CAF were also significantly elevated in patient with muscle wasting (*p* = 0.0081) A higher CAF value was present in patients who did not accomplish 400 m during 6-MWT with mean (± SD) values of CAF:123.43 ± 48.81 vs. 96.14 ± 37.54 (*p* < 0.0001) Patients who did not achieve 0.8 m/s gait speed in 4-m walk test, with mean (± SD) values of CAF: 136.73 ± 76.40 vs. 103.04 ± 39.29 (*p* < 0.003)
Scherbakov et al. 2016 Cohort study	Evaluation of C-terminal Agrin Fragment as a marker of muscle wasting in patients after acute stroke during early rehabilitation	To evaluate agrin as a marker of muscle wasting in patients with stroke	Studied 123 patients (age ranging from 42 to 98 years) with confirmed diagnosis of ischemic or hemorrhagic stroke	NR	Exclusion criteria for this observational study were acute and chronic inflammatory diseases, acute heart failure or myocardial infarction, liver cirrhosis, and dialysis, immune suppressive therapy, and history of cancer within the last 5 years	Stroke	NR	Venous blood samples were obtained under standardized conditions after overnight fasting and after 15 min of supine resting in a quiet and air conditioned room. Samples were centrifuged at 3500 rpm for 15 min (2000 × g), aliquoted and stored at − 80 °C until analysis Serum Commercially available enzyme-linked immunosorbent assay (ELISA) kit (NTCAF Elisa Kit; Neurotune, Schieren, Switzerland)	Body composition was assessed by bioelectrical impedance analysis (BIA) (QuadScan 4000, Bodystat Limited, UK)	Arm strength was analyzed using the handgrip dynamometer (Saehan Corporation, Korea)	NA	The relationship between variables was analyzed by linear and multiple regression analyses	CAF levels were elevated in stroke patients at admission (134.3 ± 52.3 pM) and showed incomplete recovery until discharge (118.2 ± 42.7 pM) compared to healthy controls (95.7 ± 31.8 pM, *p* < 0.001) Association between CAF levels and HGS (*r* = 0.2, *p* < 0.05) of the non-paretic arm
Landi et al. 2016	Serum levels of C-terminal agrin fragment (CAF) are associated with sarcopenia in older multimorbid community-dwellers: Results from the ilSIRENTE study	To determine if serum concentrations of CAF were associated with sarcopenia in a fairly large population of old and very old persons living in the community, enrolled in the “Invecchiamento e Longevità nel Sirente” (Aging and Longevity in the Sirente geographic area, ilSIRENTE) study	The mean age of the 332 participants was 86.1 years (± 1.4 SD), with a larger representation of women (n = 225; 67.7%)	All persons aged 80 years and older residing in the Sirente geographic area	NR	Multimorbid	EWGSOP	Fasting blood samples were obtained by venipuncture of the median cubital vein, using commercial collection tubes Serum Commercial ELISA kit (NTCAF ELISA, Neurotune AG, Schlieren-Zurich, Switzerland), using a Spectramax 190 UV–VIS microplate reader (Molecular Devices, Sunnyvale, CA)	The mid-arm circumference was determined with a standard flexible measuring tape	Muscle strength was assessed using a North Coast hand-held hydraulic dynamometer (North CoastMedical Inc.,Morgan Hill, CA)	Habitual walking speed was evaluated by measuring the participant usual gait speed (m/s) over a 4-m course	Analysis of covariance (ANCOVA)was performed to compare adjusted means of serum CAF levels between sarcopenic and non-sarcopenic participants	Serum levels of CAF were significantly higher in older adults with sarcopenia compared with non-sarcopenic participants (96.99 ± 5.40 pmol/L vs. 76.54 ± 2.15 pmol/L; *p* < 0.001) Serum CAF concentrations were higher in participants in the lower tertile of muscle mass (*p* < 0.001), with slower gait speed (*p* < 0.001), and lower HGS (*p* < 0.001) In women, deficits in any of the three sarcopenia domains were associated with higher circulating values of CAF (HGS: *p* < 0.03; gait speed: *p* < 0.001; muscle mass: *p* < 0.01) In men, CAF concentrations were significantly higher in those with slow gait speed (*p* < 0.05) and low HGS (*p* < 0.05)
Karim, Muhammad, Qaisar 2021	Prediction of Sarcopenia Using Multiple Biomarkers of Neuromuscular Junction Degeneration in Chronic Obstructive Pulmonary Disease	The current study aims to address this gap by evaluating circulating BDNF, GDNF, and CAF levels as potential biomarkers of sarcopenia in COPD	Male, 61–77-year-old healthy controls (*n* = 84) and patients with COPD (*n* = 77)	Subjects with stable COPD	Those with unstable COPD (infection exacerbation and/or hospitalization in the past one month), arthritis, renal or cardiac failure, prolonged bed rest, and major surgeries within the past eight weeks	COPD	EWGSOP2	NR Plasma CAF Plasma samples were analyzed using ELISA kits for CAF (NTCAF, ELISA, Neurotune, Schlieren-Zurich, Switzerland)	ASM, fat mass, and ASMI: BIA (RENPHO, Dubai, United Arab Emirates)	HGS: Digital handgrip dynamometer (CAMRY, South El Monte, CA, USA)	SPPB score	Analysis of variance was used to compare groups, and Pearson correlation was employed to determine the strength of the relationship between individual cohorts and various physical and biochemical parameters The relationship between variables was analyzed by simple and multiple regression analysis A two-sample *t test* for percent was used to compare SPPB scores among the groups	The patients with COPD had significantly higher levels of CAF (57.2% higher, *p* < 0.05) Significant negative association between CAF and HGS (*p* < 0.001) Changes in CAF vs. change in HGS (coefficient = –0.316, *p* = 0.007) Changes in CAF vs. change in ASM (coefficient –0.147, *p* = 0.071) Changes in CAF22 vs. change in Walking speed (coefficient = –0.081, *p* = 0.184)
Karim et al. 2022	Evaluation of Sarcopenia Using Biomarkers of the Neuromuscular Junction in Parkinson’s disease	To evaluate circulating CA, BDNF, and GDNF as potential biomarkers of sarcopenia in PD	142 ambulatory participants as healthy controls (*n* = 73) and patients with Parkinson Disease (PD) (*n* = 69) 63–78 years old	Participants with PD	Patients with acute or chronic inflammatory diseases, renal and cardiac failure, liver cirrhosis, major surgeries, prolonged bed rest within 8 weeks, and history of cancer within the five years were excluded	Parkinson’s Disease	EWGSOP2	NR Plasma ELISA kits for CAF (NTCAF, ELISA, Neurotune, Schlieren-Zurich, Switzerland)	Appendicular skeletal muscle mass (ASM) and fat mass were calculated with the bioelectrical impedance analysis scale (RENPHO, Dubai, UAE)	HGS was measured by a digital handgrip dynamometer (CAMRY, South El Monte, CA, USA	Short physical performance battery (SPPB) score	Analysis of variance was used to compare groups, and Pearson correlation was employed to determine the strength of the relationship between individual cohorts and various physical and biochemical parameters The relationship between variables was analyzed by simple and multiple regression analysis The areas under the curve (AUC) were calculated using receiver operating characteristics (ROC) analysis to test the utility of risk scores A two-sample *t test* for percent was used to compare SPPB scores among the groups	Patients with PD had significantly higher CAF at diagnosis (*p* < 0.0001) Patient with PD at follow up had significantly higher CAF (*p* < 0.05) CAF and SPPB scores in PD patient (9–10 score, *p* < 0.01; 6–8 score, *p* < 0.01) CAF and HGS strong correlation in PD at follow up (*r*^2^ = 0.373, *p* < 0.001) CAF and ASMI less robust correlation in PD at follow up (*r*^2^ = 0.133, *p* = 0.002) Changes in CAF vs. change in HGS (coefficient –0.269, *p* 0.015) Changes in CAF vs. change in ASMI (coefficient –0.114, *p* 0.093) Changes in CAF vs. walking speed (coefficient –0.044, *p* 0.453)

*CAF* C-terminal agrin fragment, *EWGSOP* European working group on sarcopenia in older people, *ED* emergency department, *BIA* bioelectrical impedance analyzer, *DEXA* dual-energy X-ray absorptiometry, *6-MWT* 6-min walk test, *HGS* hand grip strength, *SPPB* short physical performance battery, *COPD* chronic obstructive pulmonary disease

### Effect of interventions (exercise/nutrition/hormonal) on the levels of the CAF

A total of six studies (*n* = 6) [39, 44–48] have looked at the effect of the intervention on the level of CAF concentration. All six studies were randomized controlled trials with one (*n* = 1) randomized controlled single-blinded [39], one (*n* = 1) randomized waitlist control trial [44], one (*n* = 1) randomized placebo-controlled double-blind, parallel-group [48], one (*n* = 1) clinical trial [45], one (*n* = 1) multi-site randomized clinical trial [47], and one (*n* = 1) randomized controlled trial [46].

Different interventions were used in the included studies to determine the effect on the level of CAF. Among the six (*n* = 6) studies, only one (*n* = 1) study used testosterone therapy [48], rest five (*n* = 5) studies used exercise-based intervention [39, 44–47]. However, the mode of exercise varied between the studies, with one (*n* = 1) used resistance training [44], one (*n* = 1) used functional training (FT) with and without Blood Flow Restriction (BFR) [45], one (*n* = 1) used dual-task (DT) training with/without BFR [46], one (*n* = 1) used physical activity [47], and one (*n* = 1) used power training [39].

Resistance exercise and functional training (FT), which included the resistance exercise components, were administered in two (*n* = 2) studies [44, 45]. The frequency reported was 2–3 days/week, the intensity of 10–16 on a Borg scale/5–6 on a 10-point OMNI-Rating of perceived exertion scale, the total session time ranges from 60 to 90 min, sets and repetition ranges from 3 sets of 6–15 repetitions, and total duration of the study, 6 weeks. The physical activity program (walking, strengthening, and flexibility exercises) was used in one study (*n* = 1) with moderate intensity of 13–15 on a 20-point Borg scale with a total duration of 12 months [47]. The dual task (DT) training with and without BFR was administered in one study, with 3 days/week of frequency, respectively [46]. The DT was performed on the intensity of 45% heart rate reserve (HRR) for 8 weeks. In one of the studies, power training was given and compared to strength training among the participants who were supplemented with vitamin D [39]. The frequency was 2/week for 12 weeks, with repetition varied from 15 reps in the first week to 6 reps in the final week, while the training intensity was maintained as per the Borg rating of perceived exertion (RPE) ranging from 10 to 16 on a scale of 6–20 Borg RPE scale. Among the included six studies, only one (*n* = 1) used testosterone therapy in which testosterone doses were increased/decreased by 5 g depending on the concentration of the total testosterone till the end of the six months of treatment [48].

The body composition as an outcome measure was assessed in three studies (*n* = 3) [39, 44, 46], using DEXA in two (*n* = 2) studies [39, 46] and ultrasound imaging in one (*n* = 1) of the studies [44]. Muscle strength has been assessed in four (*n* = 4) of the studies as leg and chest press, and two (*n* = 2) used 10-repetition maximum (10-RM) as a measure of strength assessment [44, 45]. Three of the studies (*n* = 3) have assessed physical performance, with one study (*n* = 1) using a 400-m walk test and SPPB [47], a 12-step stair-climb test in one (*n* = 1) study [48], and 6-MWT in one (*n* = 1) study [46]. Two of the studies (*n* = 2) have assessed mobility as part of physical performance using the timed-up and go test (TUG) [45, 46], while only one (*n* = 1) study has not assessed the physical performance [44].

Participants were asked to undertake overnight fast in four (*n* = 4) studies [44–46, 48]. The median vein has been reported in three (*n* = 3) studies as a source from where blood was drawn [44–46]. The drawn blood sample after processing into serum in five studies (*n* = 5) [39, 44, 45, 47, 48] and plasma in only one study (*n* = 1) [46] was stored at − 80 degree Celsius in one of the studies [39], while at − 20 degree Celsius in two (*n* = 2) studies [45, 46]. Only one study (*n* = 1) used western blotting to analyze the levels of CAF [39], while three (*n* = 3) studies [44, 47, 48] and two (*n* = 2) studies [45, 46] utilized commercially available ELISA kit Neurotune and Zell bio ZB, respectively.

The results varied among the included studies, which looked at how the interventions affected the changes in CAF concentrations. In two studies (*n* = 2) which have delivered 6 weeks of resistance exercise, one in the form of functional training with blood flow restriction [45] and the other only resistance training [44], a significant decrease in serum CAF levels was observed in the resistance exercise group compared to the control (*F* (2,26) = 7.12, *p* = 0.003, ηP^2^ = 0.35) [45], while others showing increase in CAF by 10.4% (3.59 ± 1.45 to 4.00 ± 1.20 pg/mL) [44] in older adults. One (*n* = 1) study compared the strength and power training showed a significant reduction in CAF concentration in the group that underwent power training for 12 weeks [39]. Nutrition as a treatment has not been provided as a single intervention; instead, it was administered to the participants before the strength and power training. Physical activity intervention, including walking, flexibility, and balance training for 12 months, did not significantly reduce the serum CAF levels in older adults compared to health education [47]. Hormonal therapy has been utilized in only one (*n* = 1) study [48]; despite improvements in muscle strength and stair climbing power, no significant difference is noticed in the change in serum CAF concentration between testosterone and placebo group (effect size = -50.3 pM; 95% CI = -162.1 to 61.5 pm; *p* = 0.374).

Among the six (*n* = 6) included studies, five (*n* = 5) studies also reported the relationship between the CAF concentration and different outcome measures in addition to the effect. One (*n* = 1) study reported an inverse relationship between CAF and appendicular lean mass (ALM) (*r* =  − 0.524 male; *r* =  − 0.219 female). There was a positive correlation between the CAF levels and cross-sectional area (CSA) of vastus lateralis (*r* = 0.542) in one (*n* = 1) of the study. Regarding physical performance, the CAF level was significantly correlated with the gait speed (*r* =  − 0.151) in one (*n* = 1) study. At the same time, there was no significant correlation between CAF and SPPB (*r* =  − 0.086) in the same study. There were contrasting results in two (*n* = 2) studies, with one (*n* = 1) study found no significant relationship between CAF and muscle strength, while the one (*n* = 1) other study showed significant inverse correlations between the levels of CAF and knee extension strength (*r* =  − 0.45), chest press (*r* =  − 0.53). Only one (*n* = 1) study reported the correlation between CAF and muscle quality, which was inverse and statistically significant in the Dual-task Blood Flow Restriction (DTBFR) group (*r* =  − 0.81).

The two studies (*n* = 2) have reported the CAF values pre- and post-intervention. The pre–post change in the CAF values was not similar among the individuals with lower and higher baseline CAF values. In one (*n* = 1) study, at time point 1 (T1), the low CAF value was 3.14 ± 0.84, which after 12 weeks of training was found to be 3.40 ± 0.84, whereas among individuals with high CAF at time point 1 (T1) 5.69 ± 0.88, the level changed to 4.78 ± 1.77 after 12 weeks of training. The effect of resistance training was compared to the control group in one (*n* = 1) of the study; the pre-intervention CAF level in the exercise group was 3.59 ± 1.45, and in the control group, it was 3.77 ± 1.06; post-intervention CAF level in the exercise group was 4.00 ± 1.20 and in the control group 3.79 ± 1.02 (Table 3).

**Table 3:** Effect of interventions (exercise/nutrition/hormonal) on the levels of the CAF

Author, year, design	Title	Objective	Total sample and randomization		Intervention	Exercise parameters	Control group	Supervision & Motivation strategies	Outcome measures	Blood collection CAF (Serum/Plasma) CAF Analysis	CAF values	Results of relationship	Findings
Drey et al. 2013 Randomized, controlled, single-blinded	C-terminal agrin fragment as a potential marker for sarcopenia caused by degeneration of the neuromuscular junction	To evaluate relevant influencing variables on the concentration of cleaved C-terminal Agrin Fragments in the serum of prefrail community-dwelling older adults. Furthermore, the effects on CAF concentration by vitamin D supplementation and physical exercise are investigated	*n* = 69 prefrail participants (65–94 years of age) Randomized into three arms: 1. Power training (*n* = 24) 2. Strength training (*n* = 23) 3. Control group (*n* = 22)	Strength and Power training Both training groups: 5-min warm-up program of walking exercises Followed by a 20- min balance exercise program performed on the floor, on mats and on wobble boards in combination with ball-catching exercises		2/week for 12 weeks using the ‘Bodyspider’ resistance training machine (KOOPERA, Germany) Training intensity Borg’s Rate of Perceived Exertion (RPE) to 10–11 in the first weeks, Borg’s RPE to 16 in the final weeks) and reducing repetitions (15 repetitions in the first weeks, 6 repetitions in the final weeks) Both exercises included an explosive concentric phase in the PT group (fast speed) and normal speed in the ST group Each training session lasted 60 min Warm up, balance program and resistance training The exercises in the PT and ST groups were as follows: chest press, hip extension/flexion while standing, hip adduction/abduction while standing, tiptoe raises and chair rise All exercises except the last two were performed on the resistance training machine The tiptoe and chair rise movements were performed without weights with maximum repetition	All study members (including the control group) were instructed to maintain their current level of physical activity throughout the study period During the intervention phase, the control group was invited for two lectures about physical activity and healthy nutrition To improve compliance, the control group was offered to participate in a combined ST and PT program of 12 weeks after the end of the study	Supervised by trained instructors and the compliance was recorded using exercise diaries To ensure the required movement velocity, the participants were verbally encouraged	Body composition was assessed using a dual-energy X-ray absorptiometry (DXA), CAF concentration: Western Blotting	Participant’s serum samples were stored in 2 ml screw-cap polypropylene tubes and frozen at − 80 °C Serum CAF concentrations were measured using known concentrations of recombinantly produced CAF by Western blotting	Exercise Low CAF at T1: 3.14 (0.84), High CAF at T1: 5.69 (0.88) Exercise Low CAF at T2: 3.40 (0.84), High CAF at T2:4.78 (1.77)	CAF concentration and aLM (male: *r* = − 0.524, *p* = 0.015; female: *r* = − 0.219, *p* = 0.140) CAF concentration and age (male: *r* = 0.349, *p* = 0.121; female: *r* = 0.331, *p* = 0.023)	Significant reduction in CAF concentration in the group with initially high CAF concentrations The training mode itself merely shows the influence by trend (*p* = 0.077 ≤ 2α) on the change in CAF concentrations Exercise: Low CAF T1-T2: − 0.28 (1.04), Exercise: High CAF T1-T2: 0.93 (1.50), *p* value 0.004 Analysis exhibits by trend (*p* = 0.089 ≤ 2α, not shown here) that the reduction in CAF concentration is stronger in the power training group than in the strength training group
Fragala et al. 2014 randomized, wait-list controlled pilot study	Biomarkers of muscle quality: N terminal propeptide of type III procollagen and C-terminal agrin fragment responses to resistance exercise training in older adults	The purpose of this pilot study was to examine P3NP and CAF changes in response to a short-term resistance exercise program to determine if changes reflect muscle qualitative adaptations	*n* = 23 healthy older men and women (aged 61 to 85 years) volunteer to participate Participants randomized to two groups: 1. Resistance training group 2. Control group	Resistance training program: Short term, 6 week strength training program		2 workouts/week, session time 1 to 1 1/2 h, training program individualized, periodized, fully body program including exercise of various progression of all the major muscle group Included exercises: Progression of squat, split squat, leg curl, leg extension, push-up, triceps extension, calf raise, lat pull down, seated low row, biceps curl, shoulder press, abdominal plank, and reverse crunch Each workout session: Dynamic warm-up with consisting of body weight squat, high knee walking, and limb rotation terminated with an appropriate cool down 3sets, 8–15 reps, 7–8 exercises, submaximal intensity (perceived exertion not to exceed 5–6 on the ten-point OMNI scale) (approx. 70–85% of RM), 60 s of rest allotted between sets and exercises	To maintain their normal daily activities during the 6-week wait-list control and began the exercise program following post testing	Supervised resistance exercise training by a certified strength and conditioning specialist	Skeletal muscle imaging Anthropometrics: BMI and height Skeletal muscle ultrasound: Cross-sectional and echo intensity of vastus lateralis and rectus femoris DEXA: Total and regional body composition evaluated Muscle strength: Maximal voluntary dynamic leg extension strength (PLLE Power lift extension machine) Perceived exertion monitored using OMNI scale Muscle quality: Determined as relative strength (leg ext strength in KGs/lean quads muscle mass in Kgs)	Resting blood samples were collected in the morning following an overnight fast. Blood was drawn from forearm vein Serum Commercially available ELISA kit (Neurotune Catalog No: NT1001)	Pre Exercise group (*n* = 12) CAF (pg/mL) 3.59 ± 1.45 Control (*n* = 11) CAF (pg/mL) 3.77 ± 1.06 Exercise group Men: CAF: 3.78 ± 1.76 Women: CAF: 3.33 ± 1.01 Control group Men: CAF: 3.02 ± 0.38 women: CAF: 4.39 ± 1.08 Post Exercise group (*n* = 12) CAF (pg/mL) 4.00 ± 1.20/ Control (*n* = 11) CAF (pg/mL) 3.79 ± 1.02 Exercise group Men: CAF: 4.19 ± 1.54 Women 3.74 ± 0.46 Control group: Men: CAF: 3.14 ± 0.39, women: CAF: 4.32 ± 1.08 Mean change in exercise group: Men 0.1 ± 0.8 Women 0.41 ± 0.91 Mean change in control group: Men 0.12 ± 0.44 Women: − 0.07 ± 0.24	Positive correlation between change in circulating CAF and change in CSA of VL (*r* = 0.542, *p* = 0.008) where those who increased circulating CAF tended to increase CSA of the VL over the 6 weeks While those with lower baseline levels tended to lose muscle size No significant relationship between circulating CAF and muscle strength or quality change were observed	At baseline, women (3.91 ± 1.12 pg/mL) had somewhat higher circulating CAF than men (3.47 ± 1.37 pg/mL) Circulating CAF concentration increased by 10.4% (3.59 ± 1.45 to 4.00 ± 1.20 pg/mL) in older adults following 6 weeks of resistance training compared with a 0.3% observed mean change in the control group, the change as a result of exercise intervention was not statistically significant Circulating CAF appears to increase in response to short-term resistance exercise training in older adults to a clinically meaningful magnitude
Bondc et al. 2015	Effects of a one-year physical activity program on serum C-terminal Agrin Fragment (CAF) concentrations among mobility-limited older adults	To determine the effect of a one-year PA intervention on changes in circulating CAF concentrations and to evaluate association between CAF concentrations and indices of physical function	*n* = 424. Data from 333 person included in this study Physical activity (PA) and successful aging (SA) arms at 4 sites	Physical activity intervention: PA intervention include walking, strength, flexibility and balance training in center and home-based settings		Moderate intensity exercise (BORG scale) Asked to walk at target intensity of 13 (somewhat hard) and perform strength training at an intensity of 15 to 16 (hard)	SA intervention: Provide attention and health education, first 26 weeks weekly classes then monthly till end Workshop on topic like nutrition, medication use, foot care and preventive medicine Concluded by short instructor let program of upper extremity stretching exercises	Staff monitored the volume and intensity of exercise by recording the completed walking time and overall RPE each session To encourage participation: regular telephone contact	Primary: 400-m test and SPPB	Stored blood sample at baseline, 6 month and 12 month from a total of 333 participants Serum Commercially available ELISA (NTCAF Elisa Kit; Neurotune, Schlieren, Switzerland)	NA	Gait speed significantly correlated with baseline CAF level (*r* = − 0.151, *p* = 0.006) SPPB not significantly correlated with baseline serum CAF level (*r* = − 0.086, *p* = 0.115)	Significant age*time effect (*p* = 0.016): older age was associated with greater increase in serum CAF concentrations from base line to 12 months No significant group*time interaction (*p* = 0.265) one-year PA program did not significantly reduce serum CAF levels compared to health education. Sex* time did not suggest sex-related differences in response to intervention (*p* = 0.167) Indeed, the standardized beta coefficients for both baseline concentrations of and changes in circulating CAF were small (range: − 0.06 to 0.01) and thus did not significantly contribute to the final model
Gagli no Jucá et al. 2018, Randomized, placebo-controlled, double-blind, parallel-group trial	Testosterone does not affect agrin cleavage in mobility-limited older men despite improvement in physical function	To evaluate if testosterone-induced improvements in muscle strength and physical function were due to a testosterone-induced stabilization of the NMJ	*n* = 209 men screened, *n* = 99 men (46 in testosterone group) Participants were randomly assigned to receive 10 g of a transdermal gel containing either placebo or 100 mg of testosterone	Testosterone therapy		Two weeks after randomization, testosterone levels were measured and daily doses were increased or decreased by 5 g of gel if the mean TT concentration was below 500 ng/dL or above 1000 ng/dL, respectively The adjusted dose was maintained until the end of treatment, 6 months after the initiation of therapy	Placebo	NR	Strength: Leg press 1-RM using pneumatic resistance machines (Keiser Sport, Fresno, CA, USA) Chest press Physical function: 12-step stair-climb test	Fasting morning samples were drawn at baseline and at weeks 12 and 24 Serum Commercial ELISA kit (NTCAF ELISA, Neurotune AG, Schlieren-Zurich, Switzerland) Spectramax 190 UV–VIS microplate reader (Molecular Devices, Sunnyvale, CA, USA	Mean serum CAF concentration were higher in the TOM trial participants (189 pM) and were similar between groups at baseline	NR	No significant difference in the change in serum CAF concentration between testosterone and placebo group (effect size = − 50.3 pM; 95% CI − 162.1 to 61.5 pM; *p* = 0.374) Changes in CAF levels were not associated with changes in total (*p* = 0.670) or free testosterone levels (*p* = 0.747) Improvement in leg strength was not associated with a significant reduction in serum CAF levels (*p* = 0.224) Improvement in chest press strength also was not associated with a significant decrease in serum CAF concentration (*p* = 0.970) Improvement in loaded stair climbing power was not associated with a significant reduction in serum CAF levels (*p* = 0.316) Testosterone treatment did not reduce serum CAF concentrations in mobility-limited older men who otherwise showed improvement in maximal voluntary muscle strength and stair climbing power
Bigdeliet al. 2020, Clinical trial	Functional training with blood occlusion influences muscle quality indices in older adults	To determine if functional training with blood flow restriction (BFR) has a greater effect on muscle quality indices and performance of older men when compared to functional training without BFR	*n* = 30 Participants randomized into: 1. FT 2. FTBFR 3. C	The functional exercises were designed in a form of circuit training which was consisted of the following eleven stations: 1. Dumbbell fly on a Swiss ball 2. Wall squat with a Swiss ball 3. Triceps extension while lying on a Swiss ball 4. Forward lunge on a Bosu ball 5. Shoulder press while standing on a Bosu ball 6. Medicine ball squat throw 7. Standing biceps curl with dumbbells on a Bosu ball 8. Leg curl with a power band while lying on a Bosu ball 9. Seated row with power bands on Bosu 10. Crunches with a medicine ball 11. Medicine ball hyperextension from the ground The BFR group wore 5 cm pneumatic cuffs (Ghamat pooyan, Tehran, Iran) on the proximal portion of extremities. The cuffs were inflated with a manual pump, and after reaching to target pressure displayed on a gage, both pump and the gage were detached from the cuffs. The cuffs remained inflated during each set and were deflated during the rest periods between sets The order of exercises alternated between upper body and lower body		Participants in FT and FTBFR groups completed 3FT/week, for 6 weeks Each training session started with a warm-up consisted of 5 min walking on a treadmill followed by 5 min stair climber at a speed of participants’ choice Participants performed 10 reps/set in each station, then received one-minute recovery time to reach the next station Weeks 1–2: 2 sets Weeks 3–4: 3 sets Weeks 5 − 6: 4 sets In BFR group: Weeks 1–2: The cuff pressure in training sessions was 50% of the calculated AOP (∼210 − 250 mmHg for lower body and 105 − 130 mmHg for upper body) Weeks 3–4: 60% Week 5–6: 70% Training intensity: Rate of perceived exertion (RPE)	Participants in group C maintained their lifestyle	Supervision of an independent qualified fitness coach	Height: Standard Stadiometer Mobility: TUG, Balance: Modified Romberg test Flexibility: CSR Strength:10RM: B/L chest press and Knee extension	After 8 − 10 h overnight fasting, a medical laboratory scientist collected the blood samples between 0800 and 0900 from an antecubital vein into 5 cc Ethylenediaminetetraacetic acid (EDTA) tubes. Samples were spun at 3000 rpm in a 4 °C centrifuge for 10 min and separated serum was stored in a − 20 °C freezer for later analysis Serum Enzyme-Linked Immunosorbent Assays (ELISA) (ZellBio, ZB-13971C-H9648, Veltlinerweg, Germany, and ZellBio, ZB-22358C-H9648, Veltlinerweg, Germany, respectively)	The results of t-tests indicated that the circulatory levels of CAF increased significantly by 26.8% in FTBFR group, *t*(9) = 5.1, *p* = 0.001, and 19.4% in FT group, *t*(9) = 3.3, *p* = 0.009. However, 1.4 % increase in circulatory CAF in C group was not statistically significant *t*(9) = 0.4, *p* = 0.70	The results of Pearson’s correlation coefficient showed significant inverse correlations between the level of CAF and knee extension (*r* = − 0.45, *p* = 0.013), chest press (*r* = − 0.53, *p* = 0.004), static balance (*r* = − 0.51, *p* = 0.004), and positive correlation with dynamic balance (*r* = 0.45, *p* = 0.012)	The results of an ANCOVA indicated that, after accounting for the effects of the initial level of CAF, there was a statistically significant difference between groups at the end of a 6-week intervention period, with CAF being significantly lower in FTBFR group than C group *F*(2,26) = 7.12, *p* = 0.003, ηP^2^ = 0.35 However, the circulatory levels of CAF in FT group was not significantly different from FTBFR and C groups (*p* > 0.05) A significant decrease in serum C-terminal Agrin Fragment (CAF) levels were observed in FT and FTBFR groups (*p* ≤ 0.05) In addition, the levels of CAF in FTBFR group was significantly lower compared to control group
Kargaran et al. 2021, Randomized controlled trial	Effects of dual-task training with blood flow restriction on cognitive functions, muscle quality, and circulatory biomarkers in elderly women	Effects of an eight-week dual-task training with and without blood flow restriction (BFR) on muscle quality (MQ) biomarkers, physical and cognitive functions in older women	Twenty-four healthy volunteers (age: 62.9 ± 3.1 years) Participants randomized into: 1. DT with blood flow restriction (DTBFR) group (*n* = 8) 2.Dual-task (DT) group (*n* = 8) 3.Control group (C) (*n* = 8)	Participants of both experimental groups completed 24 workout sessions in eight weeks Each session included a 20-min walk on a treadmill while performing cognitive tasks Participants wore a heart rate monitor in each training session The treadmill speed was increased gradually to reach the target HR, and once the target HR was stable after 2–3 min, the speed of the treadmills was maintained for the entire training session While walking on a treadmill, participants performed several cognitive tasks All cognitive tasks, which were simple activities, were performed in the form of a two-person game and competition and aimed to increase concentration, stimulate recall abilities, and enhance mental function		3/week, Intensity 45%HRR (Heart rate reserve) At the end of each training session, the rate of perceived exertion (RPE) was assessed using a Borg scale	Control group continue with their everyday life	NR	Appendicular lean mass (aLM: DEXA (Stratos dR, DMS, France) technologies from a whole-body scan Muscle strength: One repetition maximum (1-RM) for leg extension and preacher biceps curl was estimated based on a 10-RM Aerobic fitness: 6MWT Functional ability: The timed-up and go (TUG) and Chair stand	Blood samples were collected after 8–10 h of overnight fasting from the antecubital vein 48 h before and after the intervention Blood samples were collected in two-5 cc Ethylenediaminetetraacetic acid (EDTA) tubes and were spun for 10 min at 3000 rpm in a 4 ◦C refrigerated centrifuge The plasma was separated and stored at -20 degrees C for later analysis Plasma Samples were analyzed for concentration of CAF (ZellBio, ZB- 13971C–H9648, Veltlinerweg, Germany)	The circulatory level of CAF concentration in DTBFR was significantly lower than DT (*p* = 0.009) and C groups (*p* = 0.001)	Bivariate Pearson’s correlation coefficient (*r*) results showed that the correlation between CAF and MQ was inverse and statistically significant in the DTBFR group (*r* = − 0.81, *p* = 0.015), but this relationship did not reach the significance level in the DT group (*r* = − 0.54, *p* = 0.164) and the C group (*r* = − 0.49, *p* = 0.211)	There was no significant difference between groups in circulatory blood biomarkers at baseline (*p* > 0.05) However, there was a significant interaction effect CAF (F2,21 = 14.9, *p* < 0.001, ηp^2^ = 0.59) Greater decrease in circulatory levels of CAF was observed in the DTBFR group compared to the DT group after the 8-week training intervention

*CAF* C-terminal Agrin Fragment, *RPE* rating of perceived exertion, *PT group* power training group, *ST group* strength training group, *VL* vastus lateralis, *BMI*: body mass index, *OMNI* OMNI-rating of perceived exertion scale, *TUG* timed-up and go test, *CSR* chair sit and reach, *B/L* bilateral, *RM* repetition maximum, *FTBFR* functional training blood flow restriction, *DTBFR* dual-task blood flow restriction

## Discussion

This review aimed to scope the scientific evidence about the relationship between the level of CAF and primary sarcopenia, CAF and secondary sarcopenia, and the effect of various interventions (exercise/nutrition/hormonal) on the change in the level of CAF. Many studies have been published fulfilling the eligibility criteria for the review, with an apparent surge in research about CAF concentration and its subsequent role in causing sarcopenia. The results support the hypothesis made for the higher level of CAF in primary and secondary sarcopenia. However, the results for the hypothesis that the interventions (exercise/nutrition/hormonal) will reduce the level of CAF among older adults are mixed and inconclusive.

### CAF levels among individuals with primary sarcopenia

There is variability between the included studies looking at the relationship of CAF and sarcopenia in healthy individuals, given that the studies did not use uniform diagnostic criteria for sarcopenia. Studies have reported that the individuals in the sarcopenic group have a higher concentration of circulating CAF than the non-sarcopenic group. It has been reported in the cross-sectional cohort study that CAF has a sensitivity of 61.3% and specificity of 51.7% for sarcopenia [14]. However, among the component triad of sarcopenia assessment, reduced muscle mass is found to be significantly associated with higher CAF concentration followed by reduced grip strength with more consistent findings in males. The review findings highlight the subgroup of patients with sarcopenia having higher CAF concentration with a primary mechanism of muscle wasting. Observations from the study show that CAF levels are associated with appendicular lean mass and are significantly elevated in participants with muscle wasting [49]. The sensitivity and the specificity of CAF for the low appendicular lean mass (43.3% and 70%) and low muscle strength (56.7% and 52.1%) have been reported [14]. This review also highlights the influence of gender on CAF levels, with males, irrespective of the presence of sarcopenia or not, having higher CAF concentrations compared to females. While differences in muscle architecture and adaptation mechanisms are well-established between sexes [50, 51], knowledge surrounding such disparities in biomarkers remains elusive. The person’s age also affects the level of the CAF, with higher age associated with a higher tertile of CAF [52].

Regarding the quality of the studies, four (*n* = 4) studies have clearly defined the inclusion criteria of the sample, described the study subject and setting in detail, measured the exposure in a valid and reliable way, used the standard criteria for measurement of the condition, measured the outcome in a valid and reliable way, and have used appropriate statistical analysis. Three studies (*n* = 3) have identified confounding factors and stated the strategy to deal with the same, while only one (*n* = 1) study has not reported the confounding factors and the strategy used to deal with them. The methodological quality of studies included in this section is of a good standard and shows the rigor of the researchers presenting reliable results (Supplementary material 2).

### CAF levels among individuals with secondary sarcopenia

There exists heterogeneity among the studies finding the association between the CAF concentration and secondary sarcopenia as the diagnostic criteria for sarcopenia and the population studies differed; hence, pooling the result was difficult. The population was heterogeneous with various co-morbidities, hip fracture, stroke, COPD, CHF, Parkinson’s disease, and multi-morbidities. The studies included in this review have reported that the CAF concentration levels were higher in comorbid sarcopenic individuals. Studies among individuals with CHF and multi-morbidity have reported that muscle wasting is associated with significantly elevated CAF concentration. In line with the findings of this review, studies have reported the loss of muscle mass within two months of hip fracture repair [53]. Also, in a mouse model of neuro-trypsin over-expression, the full phenotype of muscle wasting has been reported [54]. In this context, it should be noted that agrin-dependent sarcopenia can be distinguished from aging-associated muscle wasting [55]. Studies conducted on individuals with stroke, COPD, PD, and multi-morbidity in this review reported that CAF showed the strongest correlation with HGS, also the CAF concentration was significantly higher in individuals with low hand grip strength. Similar findings were reported by a study done in older women with hip fractures, that a decline in muscle strength predicted poor mobility recovery [56]. The physical performance has been measured in the studies included in this review using different tests like 6-MWT, gait speed test, and SPPB. As a third important component in assessing sarcopenia, studies included in this review reported physical performance significantly associated with higher concentrations of CAF. Similar findings have been reported in a study on mobility-limited older adults with slow-walking speed [27, 28, 47]. This review has identified a gender difference in the CAF concentration, as evident from the higher level of CAF concentration in males with comorbidity compared to females. Though reduced ALM, HGS, and physical performance are associated with higher CAF levels, the strongest association is found to be of reduced HGS, followed by physical performance and ALM in secondary sarcopenics. Musculoskeletal disease like disuse atrophy is significantly associated with higher levels of CAF [57]. The increased levels of CAF have also been reported in studies conducted on diabetic [52] and diabetic nephropathy patients [58]. A recent study examining the potential causes of cachexia in colorectal and pancreatic cancer patients discovered that pre-cachectic and cachectic patients had higher CAF concentrations than age-matched controls [59].

Regarding the quality of the studies, four (*n* = 4) studies have clearly defined the inclusion criteria of the sample, described the study subject and setting in detail, measured the exposure in a valid and reliable way, used the standard criteria for measurement of the condition, measured the outcome in a valid and reliable way, and have used appropriate statistical analysis. The confounding factors have been identified in two (*n* = 2) studies; in three (*n* = 3) studies, it is unclear, while one (*n* = 1) study did not identify confounding factors. However, the strategies to deal with the confounding factors have been reported in the statistical analysis in five (*n* = 5) of the studies, while in only one (*n* = 1) study, strategies have not been reported (Supplementary material 3).

### Changes in levels of CAF in response to interventions

In this review, studies have been included which have used the interventions to evaluate whether there could be a reversal of the degradation of the NMJ by looking at the change in the corresponding CAF levels. It was interesting to note that all the studies have provided the intervention details, allowing replication in future research. However, variability in the exercise prescription in terms of the frequency ranging from 2 to 3 times per week, intensity ranging from 10 to 16 on the Borg scale/5–6 on a 10-point OMNI-rating of perceived exertion scale, duration per session ranging from 60 to 90 min, and also the overall duration of the intervention ranging from 6 weeks to 12 months has been noticed. Currently, there is a lack of consensus among practitioners about the dosage ranging from 3 to 4 sets of 6–15 repetitions, as well as the mode of intervention (resistance training/power training/aerobic training) that is required to get the best results in terms of reduction in the level of CAF and improvement in the triad of sarcopenia outcomes of muscle mass, muscle strength, and physical performance. Two of the studies in this review have reported the non-traditional exercise, a BFR, which is a non-conventional mode of training [45, 46].

Interestingly, the findings of the included studies varied, with few reporting a decrease in the level of CAF concentration [39, 45, 46], with few reporting no change [47, 48], while surprisingly, one study reported an increase in the CAF concentration [44] following the intervention. The interventions like power training (with prior supplementation with vitamin D), FTBFR, and DTBFR decreased the CAF concentration. Similar results have been found in the studies showing that the active older adults involved in dancing present with lower CAF levels than their physically inactive peers [60]. Also, among two elderly groups undergoing training for 6 months, the group practicing dance presented decreased CAF levels, whereas CAF levels were unchanged in the group indulging in a general fitness program [29]. Reduction in CAF levels has been reported among postmenopausal women following 10 weeks of resistance training [61]. Conversely, it was noted that a 6-week resistance training intervention significantly increased the CAF concentration [44]. A similar increase in CAF levels following 10 weeks of resistance exercise has been reported in perimenopausal women [61]. This would be attributed to the increased exercise-induced stress and micro-trauma at the NMJ, which might be transient and go off with time. In contrast, the one-year physical activity program did not reduce the serum CAF concentration despite improving physical function [47]. Similarly, following the hormonal testosterone therapy, there was no change in the CAF concentration compared to the placebo despite improving muscle strength and stair climbing power [48]. Similar findings have been reported in obese older adults trained for 6 months with either only aerobic, only resistance, or a combination of both [62]. There was variation in levels of CAF following the three interventions indicating that exercise training was able to preserve but not improve NMJ health [62]. The role of doctors/health care professionals trained in geriatrics cannot be underestimated in promoting physical activity in older adults [63–65].

The findings showed that power training is more beneficial in reducing CAF concentration than strength. The possible reason could be that power training involves high neuromuscular activity for a short time which will have high reinnervating potential. These findings are from a study that showed that a high amount of neuromuscular activity protects against degeneration at NMJ resulting in sarcopenia [66]. The study that utilized FTBFR with various resistance exercises as part of the FT and the study that utilized only resistance exercises have shown contradictory results. As to improve the loss of strength and muscle mass, a combination of the mechanical and metabolic load is essential, which can be applied effectively using BFR during the training session [67]. These differences in findings among interventions may suggest that CAF responses to exercise may depend on the intensity/duration/volume of the training session [68].

We used CERT to evaluate and assess exercise interventions described in the included studies [36]. Out of the six (*n* = 6) studies included in this section, five (*n* = 5) [39, 44–47] studies delivered exercise as the intervention. The components like a detailed description of how the exercise program progressed, a detailed description of each exercise to enable replication, and a detailed description of exercise intervention have been mentioned in all five (*n* = 5) studies, while the way the exercise interventions were tailored is not reported in many of the studies (Supplementary material 4).

Three studies (*n* = 3) scored fair on the PEDro scale [39, 44, 47], two scored (*n* = 2) good [45, 46], and only one (*n* = 1) scored optimal [48]. Random allocation was done in all the studies (*n* = 6), while concealed allocation was done in only three (*n* = 3) studies [45, 46, 48] (Supplementary material 5).

### Strengths and limitations

First, a comprehensive systematic search strategy was performed in six databases, to identify a broad range of studies related to the topic. Second, no time restriction in the search strategy strengthens the search for literature published on the topic. Third, we followed acknowledged method recommendations for scoping reviews and did duplicate study selection and data extraction to raise validity. Also, to the best of our knowledge, this is the first study scoping the literature regarding the association of CAF level and components of sarcopenia (muscle mass/muscle strength/physical performance) and the various interventions used to influence the level of CAF.

This study has a few limitations as well. First and foremost, this review has considered only the published articles in electronic databases. Second, considered only full-text articles as abstracts, and proceedings were excluded. Third, we did not use search strategies with terms other than English, and we may have missed eligible studies in the other languages we intended to include.

### Future recommendations

This review has scoped the scientific literature about the intervention component and its effect on CAF concentration. A systematic review with a meta-analysis could be conducted to quantify which exercise program is most beneficial. Also, studies published in non-electronic databases and on gray literature could be carried out as an update to this review. The direction of future studies should be aimed at: (a) determining the most appropriate frequency, intensity, time, and mode of exercise or rehabilitation intervention to reduce CAF in older adults and (b) conducting a randomized controlled trial to look at the effect of a multicomponent intervention on the levels of CAF among sarcopenic older adults residing in long-term care settings and community-dwelling.

### Significance of this review

This review has scoped the scientific evidence and taxonomically summarized the results. Even though the causal NMJ mechanism leading to sarcopenia is beyond the scope of this review, a consistent association has been noticed between CAF and the sarcopenia triad. Though reduced ALM, HGS, and physical performance are associated with higher CAF levels, the strongest association is found between CAF and reduced muscle mass, followed by HGS and physical performance among primary sarcopenics. In contrast, the strongest association is found to be for reduced HGS, followed by physical performance and reduced ALM in secondary sarcopenics. Also, evidence points to power and resistance training compared to aerobic exercise in improving CAF levels among primary and secondary sarcopenics. The gender difference should be considered in designing the exercise intervention as the cause of muscle loss with aging in men may be associated with degeneration of the NMJ as measured by CAF. In women, sarcopenia may be more multifaceted. The findings may help physicians and researchers to target the specific component in rehabilitation to improve the health of older adults. In addition, the review has summarized the effect and components of exercise/nutritional/hormonal intervention to target CAF levels. The intervention studies included in the review have been evaluated using the CERT checklist, which would help the researcher and practitioners decide the mode and the parameters to be selected while prescribing the intervention (exercise/nutrition/hormone).

## Conclusions

In this review, we summarize the scientific information about the association of CAF with primary and secondary sarcopenia and also the effects of various interventions on CAF concentration. The CAF seems to be a good biomarker to distinguish between sarcopenic and non-sarcopenic older adults. Reduced ALM and HGS showed the strongest association with primary and secondary sarcopenia, respectively. The selection of training mode/parameters/exercises is critical in reducing the CAF levels and, eventually, managing sarcopenia.

## Supplementary Information

Below is the link to the electronic supplementary material.

Supplementary file1 (DOCX 19 KB)

Supplementary file2 (DOCX 34 KB)

Supplementary file3 (DOCX 29 KB)

Supplementary file4 (DOCX 51 KB)

Supplementary file5 (DOCX 15 KB)

## Data Availability

The datasets generated during and/ or analysed during the current study are available from the corresponding author on reasonable request.
