# Supplementary material for: Effect of targeted intervention on C-terminal agrin fragment and its association with the components of sarcopenia: a scoping review
Source: Aging Clin Exp Res. 2023 Mar 28;35(6):1161–86. doi: 10.1007/s40520-023-02396-w (PMC10200783; doi:10.1007/s40520-023-02396-w)
Supplement: Supplementary file 1 — Supplementary file1 (DOCX 19 KB) [file 40520_2023_2396_MOESM1_ESM.docx]

**Supplementary material 1**

**Search Strategy**

| **Concept** | **Keywords** | **MeSH term** | **Emtree** |
| --- | --- | --- | --- |
| Sarcopenia | sarcopeni*, handgrip, physical performance, skeletal muscle, muscle strength, muscle wasting, skeletal muscle mass, reduced skeletal muscle mass, muscle loss, muscle mass loss | "Sarcopenia"[Majr]) OR "Hand Strength"[Majr]) OR ( "Muscle, Skeletal"[Mesh] OR "Muscle Fibers, Skeletal"[Mesh] )) OR "Physical Functional Performance"[Majr] | 'sarcopenia'/exp OR 'muscle mass'/exp OR 'skeletal muscle'/mj OR 'muscle atrophy'/exp OR 'hand grip'/mj OR 'physical performance'/exp |
| Biomarker | neuromuscular junction, agrin, neurotrypsin | ("Biomarkers"[Mesh]) OR ( "Agrin"[Mesh] OR "agrin receptor" [Supplementary Concept] OR "C-terminal agrin fragment" [Supplementary Concept] ) | 'biological marker'/exp OR 'neuromuscular junction'/exp OR 'agrin'/exp |
| Older adults | aging, elderly, older age, older adult, older people, older person, senior citizens | Aged, aging | 'aged'/exp OR 'aging'/mj OR 'geriatrics'/mj |

**PubMed**

| #1 | ("Agrin"[All Fields] OR "C-terminal agrin fragment"[All Fields] OR "blood biomarker"[All Fields] OR "Neuromuscular junction degeneration"[All Fields] OR "neuromuscular junction"[All Fields] OR "neurotrypsin"[All Fields] OR "Biomarkers"[MeSH Terms] OR "Agrin"[MeSH Terms] OR "agrin receptor"[Supplementary Concept] OR "C-terminal agrin fragment"[Supplementary Concept]) AND ((humans[Filter]) AND (english[Filter]) AND (aged[Filter])) | 191,629 |
| --- | --- | --- |
| #2 | ("Sarcopenia"[MeSH Major Topic] OR "Hand Strength"[MeSH Major Topic] OR "muscle, skeletal"[MeSH Terms] OR "muscle fibers, skeletal"[MeSH Terms] OR "Physical Functional Performance"[MeSH Major Topic] OR "sarcopeni*"[All Fields] OR "reduced skeletal muscle mass"[All Fields] OR "muscle mass loss"[All Fields]) AND ((humans[Filter]) AND (english[Filter]) AND (aged[Filter])) | 43,900 |
| #3 | ("Aged"[All Fields] OR "older adults"[All Fields] OR "elderly"[All Fields] OR "senior citizens"[All Fields] OR "Aged"[MeSH Terms] OR "Aging"[MeSH Terms]) AND ((humans[Filter]) AND (english[Filter]) AND (aged[Filter])) | 2,895,247 |
| #4 | ("Agrin"[All Fields] OR "C-terminal agrin fragment"[All Fields] OR "blood biomarker"[All Fields] OR "Neuromuscular junction degeneration"[All Fields] OR "neuromuscular junction"[All Fields] OR "neurotrypsin"[All Fields] OR "Biomarkers"[MeSH Terms] OR "Agrin"[MeSH Terms] OR "agrin receptor"[Supplementary Concept] OR "C-terminal agrin fragment"[Supplementary Concept]) AND ("humans"[MeSH Terms] AND "english"[Language] AND "Aged"[MeSH Terms]) AND (("Sarcopenia"[MeSH Major Topic] OR "Hand Strength"[MeSH Major Topic] OR "muscle, skeletal"[MeSH Terms] OR "muscle fibers, skeletal"[MeSH Terms] OR "Physical Functional Performance"[MeSH Major Topic] OR "sarcopeni*"[All Fields] OR "reduced skeletal muscle mass"[All Fields] OR "muscle mass loss"[All Fields]) AND ("humans"[MeSH Terms] AND "english"[Language] AND "Aged"[MeSH Terms])) AND (("Aged"[All Fields] OR "older adults"[All Fields] OR "elderly"[All Fields] OR "senior citizens"[All Fields] OR "Aged"[MeSH Terms] OR "Aging"[MeSH Terms]) AND ("humans"[MeSH Terms] AND "english"[Language] AND "Aged"[MeSH Terms])) | 1,455 |

**Embase**

| #1 | **'sarcopenia'**/exp OR **'muscle mass'**/exp OR **'skeletal muscle'**/mj OR **'muscle atrophy'**/exp OR **'hand grip'**/mj OR **'physical performance'**/exp | 230,132 |
| --- | --- | --- |
| #2 | **'biological marker'**/exp OR **'neuromuscular junction'**/exp OR **'agrin'**/exp | 413,308 |
| #3 | **'aged'**/exp OR **'aging'**/mj OR **'geriatrics'**/mj | 3,659,430 |
| #4 | #1 AND #2 | 4,935 |
| #5 | #4 AND #3 | 864 |

**Scopus**

| #1 | ( TITLE-ABS-KEY ( {sarcopenia} ) OR TITLE-ABS-KEY ( "sarcopeni*" ) OR TITLE-ABS-KEY ( {muscle wasting} ) OR TITLE-ABS-KEY ( "reduced skeletal muscle mass" ) OR TITLE-ABS-KEY ( {hand grip strength} ) OR TITLE-ABS-KEY ( "physical performance" ) OR TITLE-ABS-KEY ( "muscle mass loss" ) ) | 55,460 |
| --- | --- | --- |
| #2 | ( TITLE-ABS-KEY ( {c-terminal agrin fragment} ) OR TITLE-ABS-KEY ( "bio*marker" ) OR TITLE-ABS-KEY ( {neuromuscular junction} ) OR TITLE-ABS-KEY ( {neurotrypsin} ) OR TITLE-ABS-KEY ( {caf} ) OR TITLE-ABS-KEY ( "biomarker" ) OR TITLE-ABS-KEY ( {agrin} ) ) | 584,839 |
| #3 | ( TITLE-ABS-KEY ( {aged} ) OR TITLE-ABS-KEY ( "aging" ) OR TITLE-ABS-KEY ( {older adult} ) OR TITLE-ABS-KEY ( {elderly} ) OR TITLE-ABS-KEY ( "senior citizen" ) OR TITLE-ABS-KEY ( "geriatric" ) ) | 6,830,835 |
| #4 | #1 AND #2 | 2,160 |
| #5 | #4 AND #3 | 1,458 |

**CINAHL**

| S1 | sarcopenia or sarcopenic or muscle weakness or muscular atrophy or muscle loss | 17,865 |
| --- | --- | --- |
| S2 | ( biomarkers or biological markers or biomarker or biological marker ) OR C-terminal agrin fragment | 109,497 |
| S3 | aged or elderly or senior or older people or geriatric | 1,147,545 |
| S4 | S1 AND S2 | 509 |
| S5 | S4 AND S3 | 273 |

**Web of Science**

| #1 | TS=("sarcopenia "OR "sarcopeni*" OR "muscle mass loss" OR "hand grip strength" OR "physical performance" OR "muscle wasting") | 40,806 |
| --- | --- | --- |
| #2 | TS=("C-terminal agrin fragment" OR "biomarker" OR "neuromuscular junction" OR "neurotrypsin") | 236,212 |
| #3 | AB=("older adult" OR "elderly" OR "ageing" OR "aged" OR "aging") | 977,757 |
| #4 | #1 AND #2 | 814 |
| #5 | #4 AND #3 | 314 |

[**ProQuest Health & Medical Complete (Proquest Medical Library)**](https://www.proquest.com/?accountid=50433)

| #1 | [(Sarcopenia -- Age-Related Muscle Wasting and Weakness) OR ft(muscle mass loss) OR ti.Exact("Sarcopenia")](https://www.proquest.com/recentsearches.recentsearchtabview.recentsearchesgridview.scrolledrecentsearchlist.checkdbssearchlink:rerunsearch/16133D30FB154EE9PQ/None?t:ac=RecentSearches) | 221,941 |
| --- | --- | --- |
| #2 | [(ti(C-terminal agrin fragment) OR ab(biomarker) OR ft(neurotrypsin)) OR mesh. Exact("Agrin" OR "Neuromuscular Junction" OR "Neuromuscular Junction Diseases")](https://www.proquest.com/recentsearches.recentsearchtabview.recentsearchesgridview.scrolledrecentsearchlist.checkdbssearchlink:rerunsearch/134B51C34F164F31PQ/None?t:ac=RecentSearches) | 125,319 |
| #3 | [(ab(older adults) OR ab(aging) OR ab(aged)) OR mesh. Exact("Aging")](https://www.proquest.com/recentsearches.recentsearchtabview.recentsearchesgridview.scrolledrecentsearchlist.checkdbssearchlink:rerunsearch/7A39AFE6EE54767PQ/None?t:ac=RecentSearches) | 344,433 |
| #4 | ((Sarcopenia Age-Related Muscle Wasting AND Weakness) OR ft(muscle mass loss) OR ti.Exact("Sarcopenia")) AND ((ti(C-terminal agrin fragment) OR ab(biomarker) OR ft(neurotrypsin)) OR mesh. Exact("Agrin" OR "Neuromuscular Junction" OR "Neuromuscular Junction Diseases")) | 7,332 |
| #5 | (((Sarcopenia Age-Related Muscle Wasting AND Weakness) OR ft(muscle mass loss) OR ti.Exact("Sarcopenia")) AND ((ti(C-terminal agrin fragment) OR ab(biomarker) OR ft(neurotrypsin)) OR mesh. Exact("Agrin" OR "Neuromuscular Junction" OR "Neuromuscular Junction Diseases"))) AND ((ab(older adults) OR ab(aging) OR ab(aged)) OR mesh. Exact("Aging")) | 794 |
