# Supplementary material for: Effect of targeted intervention on C-terminal agrin fragment and its association with the components of sarcopenia: a scoping review
Source: Aging Clin Exp Res. 2023 Mar 28;35(6):1161–86. doi: 10.1007/s40520-023-02396-w (PMC10200783; doi:10.1007/s40520-023-02396-w)
Supplement: Supplementary file 2 — Supplementary file2 (DOCX 34 KB) [file 40520_2023_2396_MOESM2_ESM.docx]

**Supplementary material 2**

**Association between levels of CAF and primary sarcopenia (n=4 studies)**

**Title:** Elevated levels of a C-terminal agrin fragment identifies a new subset of sarcopenia patients

**Author:** Hettwer et al

**Year:** 2013

|  | Yes | No | Unclear | Not applicable |
| --- | --- | --- | --- | --- |
| 1. Were the criteria for inclusion in the sample clearly defined? | ■ |  |  |  |
| 1. Were the study subjects and the setting described in detail? | ■ |  |  |  |
| 1. Was the exposure measured in a valid and reliable way? | ■ |  |  |  |
| 1. Were objective, standard criteria used for measurement of the condition? | ■ |  |  |  |
| 1. Were confounding factors identified? |  | ■ |  |  |
| 1. Were strategies to deal with confounding factors stated? |  | ■ |  |  |
| 1. Were the outcomes measured in a valid and reliable way? | ■ |  |  |  |
| 1. Was appropriate statistical analysis used? | ■ |  |  |  |

**Title:** C-terminal Agrin Fragment as a potential marker for sarcopenia caused by degeneration of the neuromuscular junction

**Author:** Drey et al

**Year:** 2013

|  | Yes | No | Unclear | Not applicable |
| --- | --- | --- | --- | --- |
| 1. Were the criteria for inclusion in the sample clearly defined? | ■ |  |  |  |
| 1. Were the study subjects and the setting described in detail? | ■ |  |  |  |
| 1. Was the exposure measured in a valid and reliable way? | ■ |  |  |  |
| 1. Were objective, standard criteria used for measurement of the condition? | ■ |  |  |  |
| 1. Were confounding factors identified? | ■ |  |  |  |
| 1. Were strategies to deal with confounding factors stated? | ■ |  |  |  |
| 1. Were the outcomes measured in a valid and reliable way? | ■ |  |  |  |
| 1. Was appropriate statistical analysis used? | ■ |  |  |  |

**Title:** Plasma C-Terminal Agrin Fragment as an Early Biomarker for Sarcopenia: Results From the GenoFit Study

**Author:** Pratt J et al

**Year:** 2021

|  | Yes | No | Unclear | Not applicable |
| --- | --- | --- | --- | --- |
| 1. Were the criteria for inclusion in the sample clearly defined? | ■ |  |  |  |
| 1. Were the study subjects and the setting described in detail? | ■ |  |  |  |
| 1. Was the exposure measured in a valid and reliable way? | ■ |  |  |  |
| 1. Were objective, standard criteria used for measurement of the condition? | ■ |  |  |  |
| 1. Were confounding factors identified? | ■ |  |  |  |
| 1. Were strategies to deal with confounding factors stated? | ■ |  |  |  |
| 1. Were the outcomes measured in a valid and reliable way? | ■ |  |  |  |
| 1. Was appropriate statistical analysis used? | ■ |  |  |  |

**Title:** Active older dancers have lower C-terminal Agrin fragment concentration, better balance and gait performance than sedentary peers

**Author:** Marcolin et al

**Year:** 2021

|  | Yes | No | Unclear | Not applicable |
| --- | --- | --- | --- | --- |
| 1. Were the criteria for inclusion in the sample clearly defined? | ■ |  |  |  |
| 1. Were the study subjects and the setting described in detail? | ■ |  |  |  |
| 1. Was the exposure measured in a valid and reliable way? | ■ |  |  |  |
| 1. Were objective, standard criteria used for measurement of the condition? | ■ |  |  |  |
| 1. Were confounding factors identified? | ■ |  |  |  |
| 1. Were strategies to deal with confounding factors stated? | ■ |  |  |  |
| 1. Were the outcomes measured in a valid and reliable way? | ■ |  |  |  |
| 1. Was appropriate statistical analysis used? | ■ |  |  |  |
