# Supplementary material for: Effect of targeted intervention on C-terminal agrin fragment and its association with the components of sarcopenia: a scoping review
Source: Aging Clin Exp Res. 2023 Mar 28;35(6):1161–86. doi: 10.1007/s40520-023-02396-w (PMC10200783; doi:10.1007/s40520-023-02396-w)
Supplement: Supplementary file 3 — Supplementary file3 (DOCX 29 KB) [file 40520_2023_2396_MOESM3_ESM.docx]

**Supplementary material 3**

**Association between levels of CAF and secondary sarcopenia (n=6 studies)**

**Title:** Serum levels of C-terminal agrin fragment (CAF) are associated with sarcopenia in older hip fractured patients

**Author:** Marzetti et al

**Year:** 2014

|  | Yes | No | Unclear | Not applicable |
| --- | --- | --- | --- | --- |
| 1. Were the criteria for inclusion in the sample clearly defined? | ■ |  |  |  |
| 1. Were the study subjects and the setting described in detail? | ■ |  |  |  |
| 1. Was the exposure measured in a valid and reliable way? | ■ |  |  |  |
| 1. Were objective, standard criteria used for measurement of the condition? | ■ |  |  |  |
| 1. Were confounding factors identified? | ■ |  |  |  |
| 1. Were strategies to deal with confounding factors stated? | ■ |  |  |  |
| 1. Were the outcomes measured in a valid and reliable way? | ■ |  |  |  |
| 1. Was appropriate statistical analysis used? | ■ |  |  |  |

**Title:** Detection of muscle wasting in patients with chronic heart failure using C-terminal agrin fragment: Results from the Studies Investigating Co-morbidities Aggravating Heart Failure (SICA-HF)

**Author:** Steinbeck et al

**Year:** 2015

|  | Yes | No | Unclear | Not applicable |
| --- | --- | --- | --- | --- |
| 1. Were the criteria for inclusion in the sample clearly defined? | ■ |  |  |  |
| 1. Were the study subjects and the setting described in detail? | ■ |  |  |  |
| 1. Was the exposure measured in a valid and reliable way? | ■ |  |  |  |
| 1. Were objective, standard criteria used for measurement of the condition? | ■ |  |  |  |
| 1. Were confounding factors identified? |  |  | ■ |  |
| 1. Were strategies to deal with confounding factors stated? | ■ |  |  |  |
| 1. Were the outcomes measured in a valid and reliable way? | ■ |  |  |  |
| 1. Was appropriate statistical analysis used? | ■ |  |  |  |

**Title:** Evaluation of C-terminal Agrin Fragment as a marker of muscle wasting in patients after acute stroke during early rehabilitation

**Author:** Scherbakov et al

**Year:** 2016

|  | Yes | No | Unclear | Not applicable |
| --- | --- | --- | --- | --- |
| 1. Were the criteria for inclusion in the sample clearly defined? | ■ |  |  |  |
| 1. Were the study subjects and the setting described in detail? | ■ |  |  |  |
| 1. Was the exposure measured in a valid and reliable way? | ■ |  |  |  |
| 1. Were objective, standard criteria used for measurement of the condition? | ■ |  |  |  |
| 1. Were confounding factors identified? |  |  | ■ |  |
| 1. Were strategies to deal with confounding factors stated? | ■ |  |  |  |
| 1. Were the outcomes measured in a valid and reliable way? | ■ |  |  |  |
| 1. Was appropriate statistical analysis used? | ■ |  |  |  |

**Title:** Serum levels of C-terminal agrin fragment (CAF) are associated with sarcopenia in older multimorbid community-dwellers: Results from the ilSIRENTE study

**Author:** Landi et al

**Year:** 2016

|  | Yes | No | Unclear | Not applicable |
| --- | --- | --- | --- | --- |
| 1. Were the criteria for inclusion in the sample clearly defined? | ■ |  |  |  |
| 1. Were the study subjects and the setting described in detail? | ■ |  |  |  |
| 1. Was the exposure measured in a valid and reliable way? | ■ |  |  |  |
| 1. Were objective, standard criteria used for measurement of the condition? | ■ |  |  |  |
| 1. Were confounding factors identified? | ■ |  |  |  |
| 1. Were strategies to deal with confounding factors stated? | ■ |  |  |  |
| 1. Were the outcomes measured in a valid and reliable way? | ■ |  |  |  |
| 1. Was appropriate statistical analysis used? | ■ |  |  |  |

**Title:** Prediction of Sarcopenia Using Multiple Biomarkers of Neuromuscular Junction Degeneration in Chronic Obstructive Pulmonary Disease

**Author:** Karim, Muhammad, Qaisar

**Year:** 2021

|  | Yes | No | Unclear | Not applicable |
| --- | --- | --- | --- | --- |
| 1. Were the criteria for inclusion in the sample clearly defined? | ■ |  |  |  |
| 1. Were the study subjects and the setting described in detail? | ■ |  |  |  |
| 1. Was the exposure measured in a valid and reliable way? | ■ |  |  |  |
| 1. Were objective, standard criteria used for measurement of the condition? | ■ |  |  |  |
| 1. Were confounding factors identified? |  | ■ |  |  |
| 1. Were strategies to deal with confounding factors stated? |  | ■ |  |  |
| 1. Were the outcomes measured in a valid and reliable way? | ■ |  |  |  |
| 1. Was appropriate statistical analysis used? | ■ |  |  |  |

**Title:** Evaluation of Sarcopenia Using Biomarkers of the Neuromuscular Junction in Parkinson’s Disease

**Author:** Karim et al

**Year:** 2022

|  | Yes | No | Unclear | Not applicable |
| --- | --- | --- | --- | --- |
| 1. Were the criteria for inclusion in the sample clearly defined? | ■ |  |  |  |
| 1. Were the study subjects and the setting described in detail? | ■ |  |  |  |
| 1. Was the exposure measured in a valid and reliable way? | ■ |  |  |  |
| 1. Were objective, standard criteria used for measurement of the condition? | ■ |  |  |  |
| 1. Were confounding factors identified? |  |  | ■ |  |
| 1. Were strategies to deal with confounding factors stated? | ■ |  |  |  |
| 1. Were the outcomes measured in a valid and reliable way? | ■ |  |  |  |
| 1. Was appropriate statistical analysis used? | ■ |  |  |  |
