# Supplementary material for: Effect of targeted intervention on C-terminal agrin fragment and its association with the components of sarcopenia: a scoping review
Source: Aging Clin Exp Res. 2023 Mar 28;35(6):1161–86. doi: 10.1007/s40520-023-02396-w (PMC10200783; doi:10.1007/s40520-023-02396-w)
Supplement: Supplementary file 4 — Supplementary file4 (DOCX 51 KB) [file 40520_2023_2396_MOESM4_ESM.docx]

**Supplementary material 4**

**CERT (Critical Evaluation of Intervention Reporting)**

| **Item category** | Item | Description | **Drey et al., 2013** | **Fragala et al., 2014** | **Bondc et al., 2015** | **Bigdeliet al., 2020** | **Kargaran et al., 2021** | **Section score** |
| --- | --- | --- | --- | --- | --- | --- | --- | --- |
| **What: materials** | 1 | Detailed description of type of exercise equipment | 1 | 0 | 0 | 1 | 1 | 3 |
| **Who: provider** | 2 | Detailed description of the qualification, expertise and /or training | 1 | 0 | 0 | 1 | 0 | 2 |
| **How: delivery** | 3 | Describe whether exercises are performed individually or in a group | 0 | 0 | 0 | 0 | 0 | 0 |
|  | 4 | Describe whether exercises are supervised or unsupervised; how they are delivered | 1 | 1 | 0 | 1 | 1 | 4 |
|  | 5 | Detailed description of how adherence to exercise is measured and reported | 1 | 0 | 0 | 0 | 0 | 1 |
|  | 6 | Detailed description of motivation strategies | 1 | 0 | 1 | 0 | 0 | 2 |
|  | 7a | Detailed description of the decision rule(S) for determining exercise progression | 0 | 0 | 0 | 1 | 1 | 2 |
|  | 7b | Detailed description of how the exercise program was progressed | 1 | 1 | 1 | 1 | 1 | 5 |
|  | 8 | Detailed description of each exercise to enable replication | 1 | 1 | 1 | 1 | 1 | 5 |
|  | 9 | Detailed description of any home program component | 1 | 1 | 0 | 1 | 0 | 3 |
|  | 10 | Describe whether there are any nonexercised components | 0 | 0 | 0 | 0 | 0 | 0 |
|  | 11 | Describe the type and number of adverse events that occur during exercise | 0 | 0 | 0 | 0 | 0 | 0 |
| **Where: location** | 12 | Describe the setting in which exercise are performed | 0 | 0 | 1 | 0 | 0 | 1 |
| **When How much: dosage** | 13 | Detailed description of exercise intervention | 1 | 1 | 1 | 1 | 1 | 5 |
| **Tailoring: what, how** | 14a | Describe whether exercise are generic (one size fits all) or tailored | 0 | 1 | 0 | 0 | 1 | 2 |
|  | 14b | Detailed description of how exercises are tailored to the individual | 0 | 1 | 0 | 0 | 0 | 1 |
|  | 15 | Describe the decision rule for determining the starting level | 0 | 1 | 0 | 0 | 0 | 1 |
| **How Well: planned, actual** | 16a | Describe how adherence or fidelity is assessed/measured | 0 | 0 | 0 | 0 | 0 | 0 |
|  | 16b | Describe the extent to which the intervention was delivered as planned | 0 | 0 | 0 | 0 | 1 | 1 |
| **Total** | Total score |  | 9 | 8 | 5 | 8 | 8 |  |

**Proforma CERT assessment form**

| Author and year: Drey et al., 2013  Title: C-terminal Agrin Fragment as a potential marker for sarcopenia caused by degeneration of the neuromuscular junction  Journal: Experimental Gerontology  Study location: Germany  Reviewer and date: | | | | | | |
| --- | --- | --- | --- | --- | --- | --- |
| Item | Description | Data extraction-details | Location (pg, URL etc) | | Yes: 1  No:0 | Reasons for rating eg, not reported or not clearly describes |
|  |  |  | Primary paper (Page, table, appendix) | Others (paper, protocol, website, URL) |  |  |
| 1 | Detailed description of the type of exercise equipment | ‘Bodyspider’ resistance training machine (KOOPERA, Germany) | Page 77 | - | 1 | - |
| 2 | Detailed description of the qualification, expertise and /or training | Trained instructors | Page 77 | - | 1 | - |
| 3 | Describe whether exercises are performed individually or in a group | Not reported |  | - | 0 | Not Reported |
| 4 | Describe whether exercises are supervised or unsupervised; how they are delivered | Supervised | Page 77 | - | 1 | - |
| 5 | Detailed description of how adherence to exercise is measured and reported | Compliance was recorded by using exercise diaries | Page 77 | - | 1 | - |
| 6 | Detailed description of motivation strategies | To ensure the required movement velocity, the participants were verbally encouraged | Page 77 | - | 1 | - |
| 7a | Detailed description of the decision rule(s) for determining exercise progression | Not reported |  | - | 0 | - |
| 7b | Detailed description of how the exercise program was progressed | The training intensity was augmented every fortnight by improving tensile strength (Borg’s Rate of Perceived Exertion (RPE) to 10–11 in the first weeks, Borg’s RPE to 16 in the final weeks) and reducing repetitions (15 repetitions in the first weeks, 6 repetitions in the final weeks) | Page 77 | - | 1 | - |
| 8 | Detailed description of each exercise to enable replication | Strength and Power training: Both training groups began with a 5-min warm-up program of walking exercises, followed by a 20- min balance exercise program performed on the floor, on mats and on wobble boards in combination with ball-catching exercises  All study members (including the control group) were instructed to maintain their current level of physical activity throughout the study period. During the intervention phase, the control group was invited for two lectures about physical activity and healthy nutrition. To improve compliance, the control group was offered to participate in a combined ST and PT program of 12 weeks after the end of the study  The exercises in the PT and ST groups were as follows: chest press, hip extension/flexion while standing, hip adduction/abduction while standing, tip toe raises and chair rise | Page 77 | - | 1 | Not Reported |
| 9 | Detailed description of any home program component | Control group were instructed to maintain their current level of physical activity | Page 77 | - | 1 | - |
| 10 | Describe whether there are any nonexercised components | Not reported |  | - | 0 | Not Reported |
| 11 | Describe the type and number of adverse events that occur during exercise | Not reported |  | - | 0 | Not Reported |
| 12 | Describe the setting in which exercise are performed | Not reported |  | - | 0 | Not Reported |
| 13 | Detailed description of exercise intervention | Participants trained 2 days a week for 12 weeks using the ‘Bodyspider’ resistance training machine (KOOPERA, Germany)  Both exercises included an explosive concentric phase in the PT group (fast speed) and normal speed in the ST group  Each training session lasted 60 min  Warm up, balance program and resistance training  The exercises in the PT and ST groups were as follows: chest press, hip extension/flexion while standing, hip adduction/abduction while standing, tip-toe raises and chair rise. All exercises except the last two were performed on the resistance training machine. The tiptoe and chair rise movements were performed without weights with maximum repetition | Page 77 | - | 1 | - |
| 14a | Describe whether exercise are generic (one size fits all) or tailored | Not reported |  | - | 0 | Not Reported |
| 14b | Detailed description of how exercises are tailored to the individual | Not reported |  | - | 0 | Not Reported |
| 15 | Describe the decision rule for determining the starting level | Not reported |  | - | 0 | Not Reported |
| 16a | Describe how adherence or fidelity is assessed/measured | Not reported |  | - | 0 | Not Reported |
| 16b | Describe the extent to which the intervention was delivered as planned | Not reported |  | - | 0 | Not reported |
| Total score |  |  |  |  | 9 |  |

| Author and year: Fragala et al., 2014  Title: Biomarkers of muscle quality: N terminal propeptide of type III procollagen and C-terminal agrin fragment responses to resistance exercise training in older adults  Journal: Journal of Cachexia Sarcopenia Muscle  Study location: Florida  Reviewer and date: | | | | | | |
| --- | --- | --- | --- | --- | --- | --- |
| Item | Description | Data extraction-details | Location (pg, URL etc) | | Yes: 1  No:0 | Reasons for rating eg, not reported or not clearly describes |
|  |  |  | Primary paper (Page, table, appendix) | Others (paper, protocol, website, URL) |  |  |
| 1 | Detailed description of the type of exercise equipment | Not reported | - | - | 0 | Not reported |
| 2 | Detailed description of the qualification, expertise and /or training | Certified strength and conditioning specialist | Page 141 | - | 0 | - |
| 3 | Describe whether exercises are performed individually or in a group | Not reported | - | - | 0 | Not reported |
| 4 | Describe whether exercises are supervised or unsupervised; how they are delivered | Supervised resistance exercise training | Page 140 | - | 1 | - |
| 5 | Detailed description of how adherence to exercise is measured and reported | Not reported | - | - | 0 | Not reported |
| 6 | Detailed description of motivation strategies | Not reported | - | - | 0 | Not reported |
| 7a | Detailed description of the decision rule(s) for determining exercise progression | Not reported | - | - | 0 | Not reported |
| 7b | Detailed description of how the exercise program was progressed | Resistance was adjusted to allow for the completion of the designated repetition range and to ensure participants were challenged to the specified perceived exertion rating | Page 141 | - | 1 | - |
| 8 | Detailed description of each exercise to enable replication | Included exercises: Progression of squat, split squat, leg curl, leg extension, push-up, triceps extension, calf raise, lat pull down, seated low row, biceps curl, shoulder press, abdominal plank, and reverse crunch  Each workout session: Dynamic warm up with consisting of body weight squat, high knee walking, and limb rotation terminated with an appropriate cool down | Page 141 | - | 1 | - |
| 9 | Detailed description of any home program component | Control group: To maintain their normal daily activities during the 6-week wait-list control and began the exercise program following post testing | Page 140 | - | 1 | - |
| 10 | Describe whether there are any nonexercised components | Not reported | - | - | 0 | Not reported |
| 11 | Describe the type and number of adverse events that occur during exercise | Not reported | - | - | 0 | Not reported |
| 12 | Describe the setting in which exercise are performed | Not reported | - | - | 0 | Not reported |
| 13 | Detailed description of exercise intervention | Resistance training program: short term, 6-week strength training program  2 workouts/week, session time 1 to 1 1/2-hour, training program individualized, periodized, fully body program including exercise of various progression of all the major muscle group  3sets, 8-15 reps, 7-8 exercises, submaximal intensity (perceived exertion not to exceed 5-6 on the ten-point OMNI scale) (approx. 70-85% of RM), 60 sec of rest allotted between sets and exercises.  The exercise program followed the recommended guidelines for older adults by the American College of Sports Medicine and the National Strength and Conditioning Association | Page 141 | - | 1 | - |
| 14a | Describe whether exercise are generic (one size fits all) or tailored | Tailored | Page | - | 1 | - |
| 14b | Detailed description of how exercises are tailored to the individual | Individualized | Page 141 | - | 1 | - |
| 15 | Describe the decision rule for determining the starting level | All participants completed two days of familiarization to become acquainted with the exercises and to establish resistance for each exercise | -Page 141 | - | 1 | - |
| 16a | Describe how adherence or fidelity is assessed/measured | Not reported | - | - | 0 | Not reported |
| 16b | Describe the extent to which the intervention was delivered as planned | Not reported | - | - | 0 | Not reported |
| Total score |  |  |  |  | 8 |  |

| Author and year: Bondc et al., 2015  Title: Effects of a one-year physical activity program on serum C-terminal Agrin Fragment (CAF) concentrations among mobility-limited older adults  Journal: Journal of Nutrition Health Aging  Study location: Florida  Reviewer and date: | | | | | | |
| --- | --- | --- | --- | --- | --- | --- |
| Item | Description | Data extraction-details | Location (pg, URL etc) | | Yes: 1  No:0 | Reasons for rating eg, not reported or not clearly describes |
|  |  |  | Primary paper (Page, table, appendix) | Others (paper, protocol, website, URL) |  |  |
| 1 | Detailed description of the type of exercise equipment | Not reported | - | - | 0 | Not reported |
| 2 | Detailed description of the qualification, expertise and /or training | Not reported | - | - | 0 | Not reported |
| 3 | Describe whether exercises are performed individually or in a group | Not reported | - | - | 0 | Not reported |
| 4 | Describe whether exercises are supervised or unsupervised; how they are delivered | Staff monitored (not clearly described) | Page 923 | - | 0 | Not clearly described |
| 5 | Detailed description of how adherence to exercise is measured and reported | Not reported | - | - | 0 | Not reported |
| 6 | Detailed description of motivation strategies | To encourage participation: regular telephone contact | Page 923 | - | 1 | - |
| 7a | Detailed description of the decision rule(s) for determining exercise progression | Not reported | - | - | 0 | Not reported |
| 7b | Detailed description of how the exercise program was progressed | Staff monitored the volume and intensity of exercise by recording the completed walking time and overall RPE each session | Page 923 | - | 1 | - |
| 8 | Detailed description of each exercise to enable replication | Moderate intensity exercise (BORG scale)  Asked to walk at target intensity of 13 (somewhat hard) and perform strength training at an intensity of 15 to 16 (hard) | Page 923 | - | 1 | - |
| 9 | Detailed description of any home program component | Not reported | - | - | 0 | Not reported |
| 10 | Describe whether there are any nonexercised components | Not reported | - | - | 0 | Not reported |
| 11 | Describe the type and number of adverse events that occur during exercise | Not reported | - | - | 0 | Not reported |
| 12 | Describe the setting in which exercise are performed | Participants were randomized into the PA and SA arms at four sites (Cooper institute, Stanford University, University of Pittsburgh, and Wake Forest University) | Page 923 | - | 1 | - |
| 13 | Detailed description of exercise intervention | Physical activity intervention: PA intervention include walking, strength, flexibility and balance training in centre and home-based settings  SA intervention: Provide attention and health education, first 26 weeks weekly classes then monthly till end. Workshop on topic like nutrition, medication use, foot care and preventive medicine. Concluded by short instructor let program of upper extremity stretching exercises | Page 923 | - | 1 | - |
| 14a | Describe whether exercise are generic (one size fits all) or tailored | Not reported | - | - | 0 | Not reported |
| 14b | Detailed description of how exercises are tailored to the individual | Not reported | - | - | 0 | Not reported |
| 15 | Describe the decision rule for determining the starting level | Not reported | - | - | 0 | Not reported |
| 16a | Describe how adherence or fidelity is assessed/measured | Not reported | - | - | 0 | Not reported |
| 16b | Describe the extent to which the intervention was delivered as planned | Not reported | - | - | 0 | Not reported |
| Total score |  |  |  |  | 5 |  |

| Author and year: Bigdeliet al., 2020  Title: Functional training with blood occlusion influences muscle quality indices in older adults  Journal: Archives of Gerontology and Geriatrics  Study location: Iran  Reviewer and date: | | | | | | |
| --- | --- | --- | --- | --- | --- | --- |
| Item | Description | Data extraction-details | Location (pg, URL etc) | | Yes: 1  No:0 | Reasons for rating eg, not reported or not clearly describes |
|  |  |  | Primary paper (Page, table, appendix) | Others (paper, protocol, website, URL) |  |  |
| 1 | Detailed description of the type of exercise equipment | Bosu, Medicine ball, Dumbbell, Swiss ball | Page 3 | - | 1 | - |
| 2 | Detailed description of the qualification, expertise and /or training | Independent qualified fitness coach | Page 3 | - | 1 | - |
| 3 | Describe whether exercises are performed individually or in a group | Not reported | - | - | 0 | Not reported |
| 4 | Describe whether exercises are supervised or unsupervised; how they are delivered | Supervised | Page 3 | - | 1 | - |
| 5 | Detailed description of how adherence to exercise is measured and reported | Not reported | - | - | 0 | Not reported |
| 6 | Detailed description of motivation strategies | Not reported | - | - | 0 | Not reported |
| 7a | Detailed description of the decision rule(s) for determining exercise progression | Training intensity: Rate of perceived exertion (RPE) | Page 3 | - | 1 | - |
| 7b | Detailed description of how the exercise program was progressed | In FT group:  Weeks 1–2: 2 sets  Weeks 3–4: 3 sets  Weeks 5−6: 4 sets  In FT-BFR group:  Weeks 1–2: The cuff pressure in training sessions was 50 % of the calculated  AOP (∼210−250 mmHg for lower body and 105−130 mmHg for  upper body)  Weeks 3–4: 60%  Week 5-6: 70 % | Page3 | - | 1 | - |
| 8 | Detailed description of each exercise to enable replication | The functional exercises were designed in a form of circuit training  which was consisted of the following eleven stations:  1. Dumbbell fly on a Swiss ball  2. Wall squat with a Swiss ball  3. Triceps extension while lying on a Swiss ball  4. Forward lunge on a Bosu ball  5. Shoulder press while standing on a Bosu ball  6. Medicine ball squat throw  7. Standing biceps curl with dumbbells on a Bosu ball  8. Leg curl with a power band while lying on a Bosu ball  9. Seated row with power bands on Bosu  10. Crunches with a medicine ball  11. Medicine ball hyperextension from the ground  The BFR group wore 5 cm pneumatic cuffs (Ghamat pooyan, Tehran, Iran) on the proximal portion of extremities. The cuffs were inflated with a manual pump, and after reaching to target pressure displayed on a gauge, both pump and the gauge were detached from the cuffs. The cuffs remained inflated during each set and were deflated during the rest periods between sets  The order of exercises alternated between upper body and lower body | Page 3 | - | 1 | - |
| 9 | Detailed description of any home program component | Participants in group C maintained their lifestyle. | Page 3 | - | 1 | - |
| 10 | Describe whether there are any nonexercised components | Not reported | - | - | 0 | Not reported |
| 11 | Describe the type and number of adverse events that occur during exercise | Not reported | - | - | 0 | Not reported |
| 12 | Describe the setting in which exercise are performed | Not reported | - | - | 0 | Not reported |
| 13 | Detailed description of exercise intervention | Participants in FT and FTBFR groups completed 3FT/week, for 6 weeks  Each training session started with a warm-up consisted of 5 min walking on a treadmill followed by 5 min stair climber at a speed of participants’ choice  Participants performed 10 reps/set in each station, then received one-minute recovery time to reach the next station | Page 3 | - | 1 | - |
| 14a | Describe whether exercise are generic (one size fits all) or tailored | Not reported | - | - | 0 | Not reported |
| 14b | Detailed description of how exercises are tailored to the individual | Not reported | - | - | 0 | Not reported |
| 15 | Describe the decision rule for determining the starting level | Not reported | - | - | 0 | Not reported |
| 16a | Describe how adherence or fidelity is assessed/measured | Not reported | - | - | 0 | Not reported |
| 16b | Describe the extent to which the intervention was delivered as planned | Not reported | - | - | 0 | Not reported |
| Total score |  |  |  |  | 8 |  |

| Author and year: Kargaran et al., 2021  Title: Effect of dual-task training with blood flow restriction on cognitive functions, muscle quality, and circulatory biomarkers in elderly women  Journal: Physiology & Behavior  Study location: Iran  Reviewer and date: | | | | | | |
| --- | --- | --- | --- | --- | --- | --- |
| Item | Description | Data extraction-details | Location (pg, URL etc) | | Yes: 1  No:0 | Reasons for rating eg, not reported or not clearly describes |
|  |  |  | Primary paper (Page, table, appendix) | Others (paper, protocol, website, URL) |  |  |
| 1 | Detailed description of the type of exercise equipment | Walk on a treadmill | Page 4 | - | 1 | - |
| 2 | Detailed description of the qualification, expertise and /or training | Not reported | - | - | 0 | Not reported |
| 3 | Describe whether exercises are performed individually or in a group | Not reported | - | - | 0 | Not reported |
| 4 | Describe whether exercises are supervised or unsupervised; how they are delivered | Supervised but not reported clearly | Page 4 | - | 1 | - |
| 5 | Detailed description of how adherence to exercise is measured and reported | Not reported | - | - | 0 | Not reported |
| 6 | Detailed description of motivation strategies | Not reported | - | - | 0 | Not reported |
| 7a | Detailed description of the decision rule(s) for determining exercise progression | The intensity of the training session was equivalent to 45% heart rate reserve, and this intensity maintained throughout the intervention period. The treadmill speed was increased gradually to reach the target HR, once achieved, the speed of treadmill was maintained for the entire session | Page 4 | - | 1 | - |
| 7b | Detailed description of how the exercise program was progressed | The complexity of cognitive tasks increased during the intervention period. For example, in the picture retention task, the number of pictures gradually increased from 3 to 9  The cuff pressure during training sessions was 50% of the calculated arterial occlusion pressure (AOP) and was increased by 10% every 2 weeks. Accordingly, the initial cuff pressure was 150-155 mmHg and increased by 15-20 mmHg every two weeks to reach 200 mmHg in the last two weeks | Page 4 | - | 1 | - |
| 8 | Detailed description of each exercise to enable replication | While walking on a treadmill, participants performed several cognitive tasks.  All cognitive tasks, which were simple activities, were performed in the form of two-person game and competition and aimed to increase concentration, stimulate recall abilities, and enhance mental function. The cognitive tasks included counting down from 300 to 0, naming objects, answer to question related to short videos clips that were played on the screen in front of participants, a Japanese word chain game in which one has to say a word starting with the last letter given by other and remind the order of pictures (animal, fruit, objects) that was played on the screen in front of participants. These tasks were identical for all participants in each training session. | Page 4 | - | 1 | - |
| 9 | Detailed description of any home program component | Not reported | - | - | 0 | Not reported |
| 10 | Describe whether there are any nonexercised components | Not reported | - | - | 0 | Not reported |
| 11 | Describe the type and number of adverse events that occur during exercise | Not reported | - | - | 0 | Not reported |
| 12 | Describe the setting in which exercise are performed | Not reported | - | - | 0 | Not reported |
| 13 | Detailed description of exercise intervention | Participants of both experimental groups completed 24 workout sessions in 8 weeks (3/week). Each session included a 20-min walk on treadmill while performing cognitive task.  Participants in DTBFR group wore pneumatic cuffs on the proximal portion of the legs. Cuff pressure was applied throughout the 20 min of walking to require a higher constant metabolic load | Page 4 | - | 1 | - |
| 14a | Describe whether exercise are generic (one size fits all) or tailored | Generic | Page 4 | - | 1 | - |
| 14b | Detailed description of how exercises are tailored to the individual | Not reported | - | - | 0 | Not reported |
| 15 | Describe the decision rule for determining the starting level | Not reported | - | - | 0 | Not reported |
| 16a | Describe how adherence or fidelity is assessed/measured | Not reported | - | - | 0 | Not reported |
| 16b | Describe the extent to which the intervention was delivered as planned | Not reported | Page 1 | - | 1 | - |
| Total score |  |  |  |  | 8 |  |
