# Supplementary material for: Effect of targeted intervention on C-terminal agrin fragment and its association with the components of sarcopenia: a scoping review
Source: Aging Clin Exp Res. 2023 Mar 28;35(6):1161–86. doi: 10.1007/s40520-023-02396-w (PMC10200783; doi:10.1007/s40520-023-02396-w)
Supplement: Supplementary file 5 — Supplementary file5 (DOCX 15 KB) [file 40520_2023_2396_MOESM5_ESM.docx]

Supplementary material 5

PEDro scale assessment of methodological quality (risk of bias) for intervention studies

| Criteria | Drey et al.,  2013 | Fragala et al.,  2014 | Bondoc et al., 2015 | Gaglino Jucá  et al., 2018 | Bigdeli et al.,  2020 | Kargaran et  al., 2021 |
| --- | --- | --- | --- | --- | --- | --- |
| Eligibility criteria | ✓ | ✓ | ✓ | ✓ | ✓ | ✓ |
| Random allocation | ✓ | ✓ | ✓ | ✓ | ✓ | ✓ |
| Concealed allocation | **×** | × | × | ✓ | ✓ | ✓ |
| Baseline comparability | ✓ | ✓ | ✓ | ✓ | ✓ | ✓ |
| Blinded participants | × | × | × | × | × | × |
| Blinded therapist | × | × | × | ✓ | × | × |
| Blinded assessor | × | × | × | ✓ | ✓ | ✓ |
| Adequate follow up | ✓ | ✓ | ✓ | ✓ | ✓ | ✓ |
| Intention to treat analysis | ✓ | ✓ | ✓ | ✓ | ✓ | ✓ |
| Between group comparison | ✓ | ✓ | ✓ | ✓ | ✓ | ✓ |
| Point estimate and variability | × | × | × | × | × | × |
| Total PEDro score | 5 | 5 | 5 | 8 | 7 | 7 |
